# High-speed mechano-active multielectrode array for investigating rapid stretch effects on cardiac tissue

**DOI:** 10.1038/s41467-019-08757-2

**Published:** 2019-02-19

**Authors:** Matthias Imboden, Etienne de Coulon, Alexandre Poulin, Christian Dellenbach, Samuel Rosset, Herbert Shea, Stephan Rohr

**Affiliations:** 1 Soft Transducers Laboratory, École Polytechnique Fédérale de Lausanne (EPFL), 2002 Neuchâtel, Switzerland; 2 Department of Physiology, University of Bern, Bühlplatz 5, 3012 Bern, Switzerland

## Abstract

Systematic investigations of the effects of mechano-electric coupling (MEC) on cellular cardiac electrophysiology lack experimental systems suitable to subject tissues to in-vivo like strain patterns while simultaneously reporting changes in electrical activation. Here, we describe a self-contained motor-less device (mechano-active multielectrode-array, MaMEA) that permits the assessment of impulse conduction along bioengineered strands of cardiac tissue in response to dynamic strain cycles. The device is based on polydimethylsiloxane (PDMS) cell culture substrates patterned with dielectric actuators (DEAs) and compliant gold ion-implanted extracellular electrodes. The DEAs induce uniaxial stretch and compression in defined regions of the PDMS substrate at selectable amplitudes and with rates up to 18 s^−1^. Conduction along cardiomyocyte strands was found to depend linearly on static strain according to cable theory while, unexpectedly, being completely independent on strain rates. Parallel operation of multiple MaMEAs provides for systematic high-throughput investigations of MEC during spatially patterned mechanical perturbations mimicking in-vivo conditions.

## Abstract (web summary)

While strain is known to affect cardiac electrophysiology, experimental systems to interrogate the effect of rapid strain cycles on cardiac tissue are lacking. Here the authors introduce a multielectrode array that can induce rapid dynamic strain cycles on cardiomyocyte strands and see effects of strain amplitude but not strain rate on impulse conduction.

## Introduction

Mechanical stimuli of appropriate size and timing disturb the electrical function of the heart through mechano-electrical coupling (MEC) mechanisms. Drastic examples include sudden cardiac death following impacts to the chest by baseballs or, conversely, resuscitation of patients undergoing life-threatening cardiac tachyarrhythmias by an appropriately administered thump to the chest^1^. Experimental investigations of the mechanisms underlying these phenomena require cardiac tissue to be subjected to strain amplitudes and strain rates that range from those encountered during the normal pump cycle of the heart to those present under pathophysiological conditions while permitting the simultaneous monitoring of electrophysiological parameters of interest. Among these, impulse conduction velocity is especially important because it is sensitive to changes in both passive and active electrical properties of cardiac tissue and, hence, is a broad indicator for strain-induced adverse electrophysiological effects on the heart that contribute to the precipitation of arrhythmias^2^. While available experimental approaches based on stepper motor^3^ or indenter technology^4^ permit stretching of cardiac tissues to physiological levels and beyond (>10–15%; for review cf. refs. ^5–8^), strain rates offered by these systems are generally substantially slower^3^ or barely exceed^4^ those encountered under physiological conditions (1–2 s^−1^, determined from changes in sarcomere lengths in intact hearts^9^). To achieve faster strain rates, we developed an experimental system (i) that is based on self-contained, mechanically active cell culture wells, (ii) that permits the reproducible application of defined levels of strain (of at least 10%) at predefined strain rates (up to 18 s^−1^ are shown but the technology has the potential to approach strain rates of 1000 s^−1^ ^10^) to cultured excitable cells, and (iii) that allows for continuous determinations of conduction velocities immediately after strain application. Experiments with bioengineered strands of rat ventricular cardiomyocytes revealed that, surprisingly, conduction velocity is independent on strain rates even if rates surpass physiological levels by an order of magnitude.

## Results

### Self-contained mechano-active multielectrode array

**Fig. 1:**
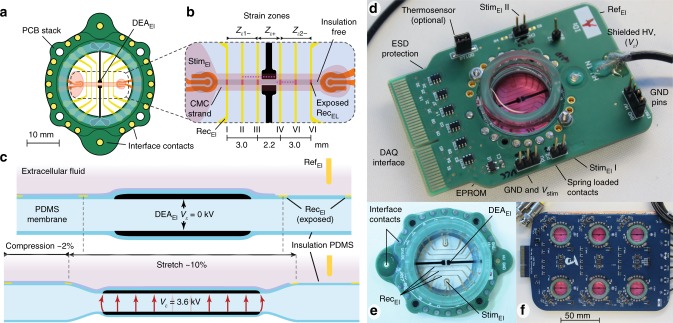
Mechanically active multielectrode array (MaMEA). **a** Schematic drawing of the culture well with the bottom being formed by a pre-stretched polydimethylsiloxane (PDMS) membrane that is anchored to two printed circuit board (PCB) frames. The top PCB interfaces with the gold extracellular electrodes (ECEs) that serve to record (recording electrodes, yellow) and stimulate (stimulation electrodes, orange) the patterned cardiac tissue (shape shaded in red). The black structures refer to the ground electrodes (carbon-loaded PDMS) of the dielectric actuator (DEA). The bottom PCB interfaces with the high-voltage DEA electrodes (carbon-loaded PDMS; same shape as ground electrodes). **b** Magnified view of the center region of the culture well: application of high voltage to the DEA electrodes results in regions exhibiting positive uniaxial strain (*Z*_*ε*+_; flanked by the two Rec_Els_, III and IV) and two adjacent zones undergoing negative strain (*Z*_*ε*−_ with Rec_Els_ I–III and IV–VI, respectively). The dark shaded area within the light-red region (tissue shape) refers to non-insulated regions where cardiomyocytes (CMCs) can make direct contact with the Stim_Els_ (indicated in red). **c** Cross-section of the membrane along the dashed line in **b** (not to scale): upon activation of the DEA, the cells in zone *Z*_*ε*+_ are stretched to either side of the center. **d** Image of the fully assembled device with the MaMEA mounted on the motherboard that connects to the DAQ. A shielded HV coaxial cable is used to supply the DEA voltage. **e** Top view of the MaMEA. **f** Different implementation of the system consisting of an array of six individually addressable MaMEAs. The motherboard includes signal amplification and conditioning electronics

An overview of the components of the mechano-active multielectrode array (MaMEA) system is presented in Fig. 1 and the operating principle of the dielectric actuator (DEA) is outlined in Supplementary Fig. 1 The modular device is built around a cell culture well whose bottom consists of a pre-stretched polydimethylsiloxane (PDMS) membrane that is sandwiched between two ring-shaped printed circuit boards (PCBs) (Fig. 1a, e). The bottom PCB includes high voltage leads for driving the dielectric elastomer actuators (DEAs), whereas the top PCB serves to route the signals from the extracellular electrodes to the interface contacts. A total of 10 linearly arranged and compliant extracellular electrodes are incorporated into the PDMS membrane using gold ion implantation techniques (Fig. 1b)^11^. The two outermost electrode pairs (Stim_Els_) are patterned such as to permit an efficient bi-polar electrical stimulation of the tissue. The six interposed electrodes (Rec_Els_, labeled I–VI) are used for recording extracellular action potentials (AP_ECs_) during propagated activity along the tissue. A thin layer of PDMS electrically insulates the Rec_Els_, with the exception of the center region which is in contact with the excitable tissue. Mechanical strain is generated by two symmetrical DEAs whose design principles were described previously^12–16^ and are outlined in Supplementary Note 1. In short, mechanical actuation is based on the electrostatic force generated by potentials applied between two stretchable carbon electrodes that are separated by a pre-stretched PDMS membrane acting as the dielectric (Fig. 1c). Upon applying an electric field, the incompressible PDMS expands laterally, which induces tensile strain on the membrane between the two sets of DEA electrodes (*Z*_*ε*+_ zone) and compressive strain to either side of this central region (*Z*_*ε*1−_ and *Z*_*ε*2−_ zones; Fig. 1b). The DEA electrode geometry was chosen such that the membrane between the two DEAs undergoes uniform–uniaxial strain. Because this region is transparent, cells can be microscopically observed in real time during strain application. Further fabrication and actuation details are given in the Methods section and Supplementary Note 2 (Supplementary Fig. 2). Interfacing of the MaMEA with the digital control and acquisition system (DAQ) and the DEA drive signals is accomplished through the motherboard (Fig. 1d) that connects to the cell culture well by means of spring-loaded pins. Because DEA actuation signals can reach up to 5 kV, all DAQ connections include electrostatic discharge (ESD) protection to prevent damage to the electronics in the case of high-voltage breakdown of the PDMS membrane. Moreover, Stim_Els_ are galvanically isolated from the Rec_Els_ in order to reduce the size of stimulation artifacts. While most of the data presented were obtained with single-well devices, a multiwell configuration that enables higher throughput experiments and includes signal amplification and conditioning electronics has been realized as well (Fig. 1f and Supplementary Note 3, with Supplementary Fig. 3 depicting the functional array configuration of the motherboard with on-board signal conditioning).

**Fig. 2:**
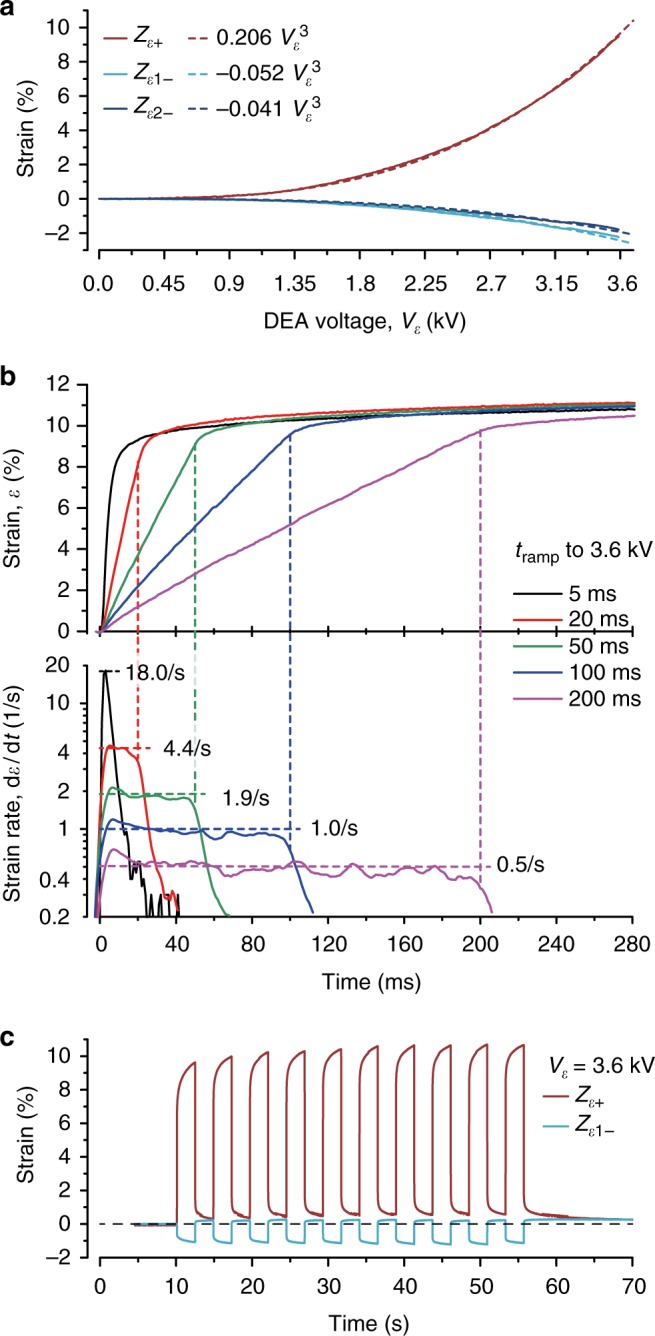
Mechanical response characteristics of the mechano-active multielectrode array (MaMEA). **a** Plot of strain in zones *Z*_*ε*+_, *Z*_*ε*1−_, and *Z*_*ε*2−_ as a function of voltage applied to the DEA, *V*_*ε*_. The fit shows a cubic relationship. **b** Strain (upper panel) and strain rate (lower panel) during the first 280 ms of linear ramps to ~10% strain (*V*_*ε*_ = 3.6 kV). The ramp time (*t*_ramp_) is varied from ~5 ms (step) to 200 ms. The resulting strain rates range from 18 to 0.5 s^−1^. **c** Example of strain profiles as used for the experiments. After a 10-s-long baseline recording, the strain is modulated with a period of 4.8 s for 10 cycles. Protocols are repeated with varying strain amplitudes and ramp times. In the example shown, *t*_ramp_ was fixed at 5 ms (corresponds to a strain rate of 18 s^−1^)

The mechanical characteristics of the MaMEA system are illustrated in Fig. 2 with Fig. 2a depicting strain (*ε*) development as a function of the applied DEA voltage (*V*_*ε*_). While the capacitive force (Maxwell pressure) acting between two fixed electrodes scales with the square of the applied voltage, we observed that *ε* in fact scales with *V*^3^ for strains up to 20%, which is explained by the thinning of the dielectric (PDMS) that accompanies increasing stretch^17^. At *V*_*ε*_ = 3.6 kV, *Z*_*ε*+_ was stretched by ~10%, while both *Z*_*ε−*_ zones (*Z*_*ε*1−_ and *Z*_*ε*2*−*_) underwent nearly symmetric compression by ~−2%. Strain rates achieved with the MaMEA during maximal stretch (*V*_*ε*_ = 3.6 kV) were measured using a high-speed camera and are depicted in Fig. 2b. According to the observation that *ε*~*V*^3^, constant strain rates were obtained by ramping the DEA voltage as cubic root with respect to time $\left( {V_\varepsilon (0 < t < t_{{\mathrm{ramp}}}) = V_{\mathrm{max}} \times \root {3} \of {{\frac{t}{{t_{{\mathrm{ramp}}}}}}}} \right)$ (a discussion of the DEA actuation and strain calibration is presented in Supplementary Note 4). Strain rates applied ranged from of 0.5 s^−1^ (ramp time, *t*_ramp_: 200 ms) to 18 s^−1^ (*t*_ramp_: 5 ms).The latter exceeds physiological strain rates (1–2 s^−1^) and approaches rates associated, for example, with trauma in neurons^18–20^. Strain protocols applied during the biological measurements of this study are shown in Fig. 2c. After a 10-s-long recording of AP propagation under control conditions, preparations were subjected to 10 cycles of identical strains lasting 4.8 s each (50% duty cycle) followed by another 10 s of baseline recording. Strain amplitudes and *t*_ramp_ were systematically changed during successive runs of this protocol that were separated by >60 s from each other. Typically, 85% of total strain was attained during the ramp, thus setting the strain rate. The remaining ~15% developed more slowly due to relaxation processes in the DEA^21^. This “creep” of strain is seen in each cycle and accumulates over the first ~5 cycles, after which the system reaches steady state. A lesser amount of creep was observed during relaxation. To account for creep effects, strain development must either be continuously monitored during the experiments or, as done in this study, devices undergo a detailed strain calibration prior or post experiments. Without real-time measurements of strain during the experiments, strain accuracy is limited by creep with an estimated uncertainty of ~5% of the full strain amplitude (i.e., an absolute error of 0.5% for 10% strain). A complete set of voltage actuation waveforms and the corresponding predicted strain profiles achieved with the MaMEA is shown in Supplementary Fig. 4.

### Assessing electrical activation with compliant electrodes

**Fig. 3:**
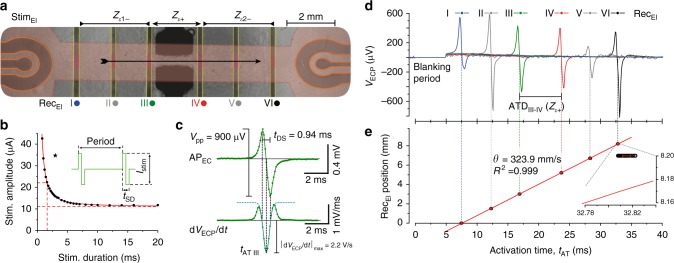
Stimulation and recording with flexible gold ion-implanted electrodes. **a** Composite image of a cell strand (red) with stimulation (orange) and recording electrodes (yellow) on either side of the DEA electrodes (black). The center arrow illustrates the direction of action potential propagation. **b** Strength–duration curve for stimulation of the preparation with bi-polar current pulses (inset). The star indicates typical stimulation parameters used during the experiments. **c** Upper panel: AP_EC_ as recorded by one electrode during propagated electrical activity along the cell strand. The AP_EC_ exhibits an amplitude (*V*_pp_) of 900 µV and, as calculated from the Gaussian fit to the first derivative (lower panel), a downstroke time (*t*_DS_) of 940 ms and a maximal downstroke velocity of −2.2 V s^−1^. The negative peak of the fit was used to determine the local activation time (*t*_AT_). **d** AP_ECs_ recorded by all electrodes during propagated electrical activity. The initial 5 ms of the signals containing the stimulation artifact were blanked. **e** Plot of local activation times vs. position for each recording electrode. Conduction velocity (*θ* *=* 324 mm s^−1^) is derived from the slope of a linear fit to the data. The insert indicates the presence of minimal *t*_AT_ variability (SD < 10μs, *n* = 170 serial stimulations) at Rec_El_ III during 170 successive measurements

Primary cultures of neonatal rat ventricular cardiomyocytes were grown on the PDMS membrane as to form a ~16-mm-long barbell with the bells covering the ion-implanted Stim_Els_ and the bar (9.7 mm long, 1.6 mm wide) covering the non-insulated regions of the Rec_Els_ that spanned both the stretch (*Z*_*ε*+_) and compression (*Z*_*ε*−_) regions of the substrate (Figs. 1a, 3a). The cardiomyocyte patterns were generated using lift-off techniques (see Supplementary Note 5 for further details). Cardiomyocyte remained firmly attached to the substrate and to the electrodes during application of strain, that is, Rec_Els_ always detected signals from the same group of cardiomyocytes irrespective of the level of strain. The functionality of the gold ion-implanted Stim_Els_ was tested by assessing the threshold for successful electrical stimulation of the preparations with bi-polar current pulses while systematically varying stimulation amplitude and duration. This resulted in a typical hyperbolic strength–duration curve with the chronaxie amounting to 2.1 ms at a stimulation strength of 11.1 µA (Fig. 3b). During experiments, cardiomyocyte strands were continuously stimulated at 2.5 Hz with bi-polar current pulses lasting 2–4 ms at double threshold intensity (typically 20 to 60 µA). Gold ion-implanted electrodes also performed well as Rec_Els_ as demonstrated by the example of an AP_EC_ recording shown in Fig. 3c. The AP_EC_ displayed a typical biphasic shape with an amplitude (*V*_pp_) of 0.9 mV. Further parameters extracted from a Gaussian fit to the first derivative of the AP_EC_ included the local activation time, *t*_AT_, (fit error of ~6µs), the maximal downstroke velocity, $\left| {\frac{{{\mathrm{d}}V_{{\mathrm{ECP}}}}}{{{\mathrm{d}}t}}} \right|_{{\mathrm{max}}}$, and the duration of the downstroke, *t*_DS_. AP_EC_ recorded from all six Rec_Els_ during action potential propagation along a cardiomyocyte cell strand from left to right are shown in Fig. 3d with the corresponding plot of *t*_AT_ vs. distance depicted in Fig. 3e (detailed analysis procedures and noise measurements are presented in Supplementary Note 6). As indicated by the linear fit of the *t*_AT_ vs. distance data (*R*^2^ = 0.999), conduction was highly uniform and exhibited an average velocity, *θ*, of 323.9 ± 0.1 mm s^−1^. The inset in Fig. 3e illustrates that the temporal spread of *t*_AT_ measured at Rec_El_VI during a total of 170 consecutive stimulations at 2.5 Hz was <20 µs. This amounts to <0.1% of the overall activation time of the preparation and demonstrates the presence of stable and highly reproducible action potential propagation under control conditions. Supplementary Note 7 characterizes the preparation stability with regards to *t*_AT_, *t*_DS_, *V*_pp_, and $\left| {\frac{{{\mathrm{d}}V_{{\mathrm{ECP}}}}}{{\mathrm{d}t}}} \right|_{{\mathrm{max}}}$ without strain modulation.

For the experiments reported in this study, field potentials recorded by the gold ion-implanted electrodes were AC coupled to the amplifiers with a coupling time constant (*τ*_c_) of 8 ms. While this setting stabilizes signal baselines and thereby permits a precise extraction of local activation times from signals caused by the fast action potential upstroke, the short time constant simultaneously suppresses field potentials arising from slower transmembrane voltage changes associated with repolarization. Supplementary Note 8 demonstrates that, accordingly, an increase of *τ*_c_ to 1600 ms unveils the repolarization-dependent signal which permits, for example, to assess frequency-dependent changes in action potential duration (APD restitution, see Supplementary Fig. 10). In the same supplementary note, we also show that the MaMEA system is suitable to record electrograms from stem-cell derived human cardiomyocytes (Supplementary Fig. 11). Both of these findings illustrate that the MaMEA system is suitable for cell types different from neonatal rat ventricular cardiomyocytes and that the system may also be used for investigating the effects of strain cycles on repolarization and action potential duration, respectively.

### Measuring impulse conduction in strained preparations

**Fig. 4:**
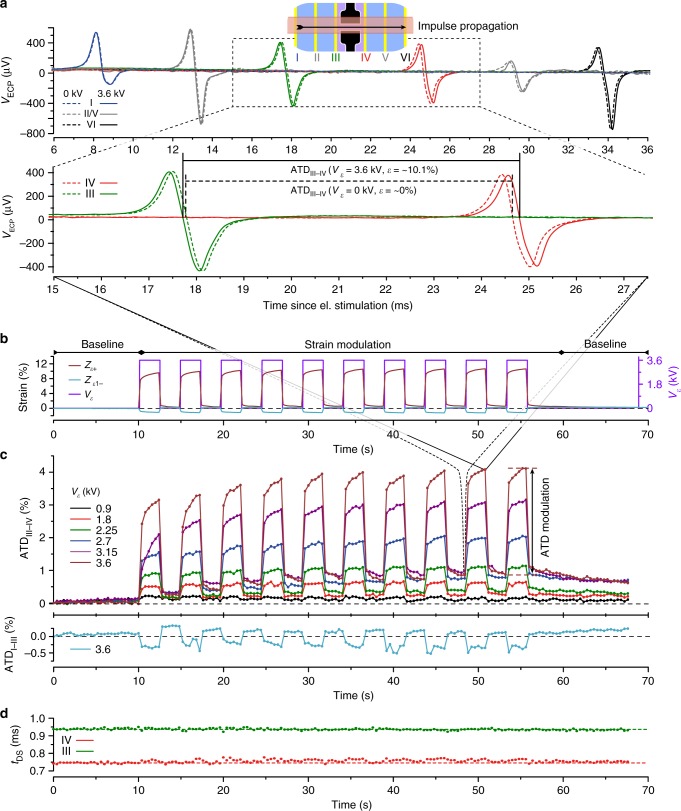
Effect of strain amplitude on impulse conduction. **a** Upper panel: AP_ECs_ recorded by electrodes I to VI during action potential propagation from left to right under control (dashed line) and strained conditions (solid line). Lower panel: Magnified view of the signals recorded by electrodes III and IV that straddle the positive strain zone show an increase of the activation time difference (ATD) by 2.7% during application of 10.1% strain. **b** Experimental strain protocol depicting the actuation voltages for the DEA (black) together with the measured response of the polydimethylsiloxane (PDMS) substrate in the positive (dark red) and negative strain (light blue) zones. **c** Modulation of ATD in the *Z*_*ε*+_ region for different strain amplitudes (*V*_*ε*_ = 1–4 kV according to color scheme); ATD decrease in the *Z*_*ε−*_ region is shown for *V*_*ε*_ = 3.6 kV only (light blue). **d** Continuous determinations of downstroke times (*t*_DS_) as recorded by electrodes III and IV at maximal strain amplitude (*V*_*ε*_ = 4 kV). All data were obtained from a single preparation

To determine the effect of strain on the impulse propagation, preparations were paced at 2.5 Hz while being subjected to the strain protocol shown in Fig. 2c. The pacing rate permitted the determination of 25 activation time differences (ATDs) between pairs of specified Rec_Els_ and, hence, between defined regions within the preparation during the initial and final baseline conditions and 12 determinations during each strain–relaxation cycle. Strain cycles were synchronized to pacing such that activation of the tissue occurred 300 ms after the onset of strain and relaxation, respectively, under static strain conditions. This allowed even the slowest ramps (*t*_ramp_ = 200 ms) to be completed before electrical activation. AP_ECs_ recorded by the six Rec_Els_ during action potential propagation under control (dashed line) and strained conditions (solid line) are shown in Fig. 4a. Strain (*V*_*ε*_ = 3.6 kV; *t*_ramp_: 5 ms) caused an early appearance of AP_ECs_ at Rec_Els_ I–III and a delayed appearance at Rec_Els_ IV–VI where the difference of the ATD recorded by Rec_Els_ III and IV, straddling *Z*_*ε*+,_ increased from 6.851 to 7.004 ms (+2.7%). Strain profiles present in the *Z*_*ε*+_ and *Z*_*ε−*_ regions during these experiments are shown in Fig. 4b. In the *Z*_*ε*+_ region, strain did not exactly mirror the actuation voltage (3.6 kV square pulses), but showed, as described above, an upward creep during DEA activation and a lesser pronounced downward creep during relaxation. An analogous behavior, albeit at reduced amplitudes and with reversed polarity, was observed in the *Z*_*ε−*_ regions. As shown in Fig. 4c, ATDs measured between Rec_Els_ III and IV closely followed the developed strain with ATDs increasing in the *Z*_*ε*+_ region by ~4% for the largest strain applied (10.0%) while decreasing by ~−0.5% in the *Z*_*ε−*_ region undergoing simultaneously compression by ~1.4%. This close correlation between strain and ATD is also evident during phases of creep that are mirrored by similarly shaped changes in ATDs. Discordance between strain and ATDs was present in the non-strained intervals where, following large strain amplitudes, the substrate showed near-complete relaxation, whereas ATDs exhibited a small but systematic increase following consecutive strain cycles (most notably for the first five cycles) and only slowly recovered during the final baseline recording period. (The AP_EC_ recordings during strain application to the cell culture and the resulting modulation of the ATD are illustrated in the Supplementary Movie 1.) By contrast to the strain-dependent changes of ATDs, *t*_DS_ of signals recorded by Rec_Els_ III and IV (Fig. 4d) were not affected by strain which suggests that active membrane properties and specifically the sodium inward current were little affected by the strain protocol used. The possibility that large electric fields associated with the activation of the DEAs may have affected conduction and, hence, ATDs, was investigated by mechanically stabilizing the substrate before applying standard strain protocols to the MaMEA. These measurements showed (Supplementary Fig. 14) that DEA activation in the absence of strain had no effect on ATDs, thereby excluding the presence of unspecific effects of electric fields on impulse conduction.

### Effects of strain dynamics on impulse conduction

**Fig. 5:**
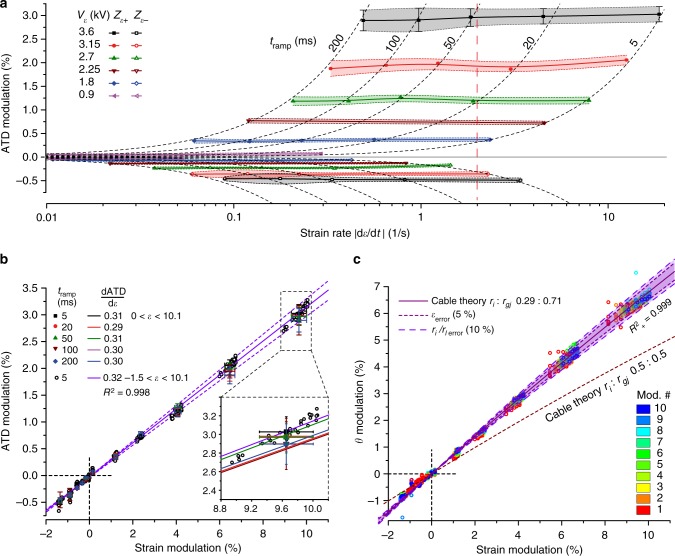
Effects of increasing strain rates and strain amplitude on impulse conduction. **a** Modulation of activation time differences (ATDs) measured across the *Z*_*ε*+_ region (above horizontal line) and *Z*_*ε−*_ region (below horizontal line) as a function of maximal strain rates and amplitudes. The shaded area (extrapolated as a B-spline from the individual measurements) illustrates the extent of creep. The dotted lines depict identical ramp times (ATD = *α ε t*_ramp_ d*ε*/d*t*). **b** ATD modulation as a function of strain amplitude and strain rates. ATD modulation correlates linearly with strain amplitude (slope *α* *=* 0.30 (*ε* > 0) and *α* *=* 0.32 (all strain values)). The inset referring to the data obtained at largest strain amplitudes demonstrates that the slope is not dependent on strain rate (*α*_mean_ = 0.302, *α*_st.dev_ = 0.008). Horizontal error bars denote variations of strain amplitudes over multiple modulations, vertical error bars denote variations of ATDs at given strain amplitudes caused by creep. **c** Dependence of the impulse conduction velocity, *θ*, on strain amplitude. Circles denote results obtained during a complete set of experiments consisting of 10 successive strain cycles. All data were obtained from a single preparation. For further experiments cf. Supplementary Fig. 13. Source data are provided^35^

To investigate whether impulse conduction in strained preparations is dependent on strain rates and amplitudes, we systematically varied strain rates by altering *t*_ramp_ from 5 to 200 ms for six strain amplitudes ranging from 0.2 to 10.0% in a single preparation. Strain-dependent modulation of ATD was determined in respect to the last ATD determination of the previous relaxation period to accommodate for creep effects. In accordance to the findings presented above (Fig. 4), ATDs in *Z*_*ε*+_ increased with increasing strain amplitude while simultaneously decreasing in *Z*_*ε−*_ (Fig. 5a).

Surprisingly, ATDs failed to show a dependence on strain rates even for the fastest rates (18 s^−1^) and largest strain amplitudes (~10.0%) tested. The same was observed for ATDs in the *Z*_*ε*−_ regions which remained unaffected by changes in strain rates and amplitudes (Fig. 5a). When plotted as a function of strain amplitude (Fig. 5b), ATDs showed a linear dependence on strain with slopes (*α*) being virtually identical for the five different strain rates tested (see inset, *α* = 0.302 ± 0.008; mean ± SD). Compression zones *Z*_*ε*−_ showed a highly similar behavior with reversed polarity and a slope of the linear fit amounting to 0.32 when including measurements from both the positive and negative strain zones. From the finding that ATDs are linearly dependent on strain, changes in true *θ* can be calculated by considering that $\frac{\theta }{{\theta _0}} = \frac{{\varepsilon + 1}}{{\alpha \varepsilon + 1}}$ (see Supplementary Note 9 for details). This function predicts a reduced dependence of *θ* on strain at large amplitudes which is indeed reflected by the data (Fig. 5c). Practically, the function indicates that *θ* increases by ~7% for a strain amplitude of 10% (321–342 mm s^−1^). Under the assumption that strain does not affect active membrane properties of the preparation, the increase of *θ* is explained by cable theory for the case that the ratio of axial resistance to gap junctional resistance is 0.29:0.71 (for details cf. Supplementary Note 10, the equivalent circuit diagram is illustrated in Supplementary Fig. 12, the scaling laws are derived in Supplementary Table 1). Experiments with five additional independent preparations being subjected to identical strain protocols produced highly similar results (cf. Supplementary Note 11 where five additional experiments are reproduced in Supplementary Fig. 13).

## Discussion

With each pump cycle, the ventricles of the human heart are subject to positive strain during isovolumetric relaxation and during early and late diastolic filling which cause cardiomyocytes to be stretched by ~13% at strain rates of 1–2 s^−1 ^^9,22–24^. At comparable maximal strain amplitudes (10%), the MaMEA presented here delivers strain rates surpassing physiological levels by an order of magnitude. The absolute precision of strain profiles reached ~95% of the total strain amplitude due to the viscoelastic properties of the PDMS membrane, which caused an upward creep of strain during activation and a downward creep during deactivation of the DEAs with an opposite behavior observed in zones of tensile compression. By contrast to this short-term effects, mechanical long-term drift was minimal and repeated strain modulations had no lasting effects on the conduction characteristics as described in Supplementary Note 7. It was observed that the baseline remained within 0.3% of the initial recording. The gold ion-implanted compliant electrodes permitted continuous measurements of extracellular potentials during actuation of the substrate. The performance of these electrodes matched that of standard rigid MEA electrodes both in terms of stimulation efficiency and shape and size of the field potentials associated with the action potential upstroke^25–27^. Importantly, AP_ECs_ recorded by compliant electrodes remained stable during experiments as illustrated by the finding that, after hundreds of strain cycles at maximal amplitude, AP_EC_ characteristics remained unchanged. This finding, together with the observation that cell strands remained firmly attached to the substrate during the experiments, implies that cell adherence to the substrate was not compromised by high strain amplitudes and high strain rates. In parallel to the assessment of uniaxial stretch on conduction, the MaMEA has the unique feature of permitting the simultaneous investigation of the effect of compression on conduction. Apart from serving as an internal control that can unmask differential responses to positive and negative strain, the apposition of electronically coupled *Z*_*ε*+_ and *Z*_*ε*+_ regions may serve as a model for diseased hearts where zones of negative and positive strains coexist as is the case, for example, in infarct borderzones^28^. Based on these particular properties of the DEA actuation, it is conceivable to construct geometrically defined strain patterns that reproduce specific patterns present in diseased hearts by appropriate redesign of the geometrical layout and number of active DEA electrodes^10^.

In the experiments presented, we fed the signals of the gold ion-implanted electrodes to the amplifiers using a fast time constant (*τ*_c_) of the AC-coupling circuit. This has the advantage of stabilizing signal baselines without compromising the action potential upstroke related fast signals used for assessing strain-dependent changes in impulse conduction. On the downside, this setting causes suppression of slower signals as those associated with action potential repolarization. As shown in Supplementary Note 8, the MaMEA system is capable of recording repolarization related signals after appropriately increasing *τ*_c_. For the triangularly shaped rat action potential, the repolarization-dependent signal followed immediately upon the initial fast signal as reported before^29^. On the other hand, preparations consisting of stem-cell derived human cardiomyocytes exhibiting a pronounced plateau phase produced a repolarization signal that was separated from the initial fast signal by the length of the plateau^30,31^. These findings demonstrate that the MaMEA system can be used with preparations different from rat ventricular cardiomyocytes and that, apart from investigating the effect of strain on conduction velocity as done in this study, the system may also be suited for the determination of the effects of dynamic strain cycles on action potential duration.

The coupling interval between completion of a given strain ramp and electrical activation in the experiments presented was systematically varied with the shortest interval amounting to 300 ms. This interval can be deliberately shortened to 2–3 ms, which corresponds to the time needed for the amplifiers to recover from saturation secondary to fast changing electrical fields during DEA activation/deactivation. Also, by using ramp times shorter than the ATD between the central two recording electrodes (~7 ms), we found in preliminary experiments that it is feasible to assess effects of very fast strain events on conduction at the very moment when activation invades the central strained region of the MaMEA (data not shown). Such close coupling between strain events and electrical activation is not only essential for reproducing pathophysiological events like an impact to the chest interfering with ongoing electrical activation of the heart, but it is prerequisite for investigations of the effects of very fast inactivating stretch-sensitive ion channels like the piezo family^32^ on impulse conduction in excitable tissues.

The results of our measurements obtained at low strain rates concurred with findings of previous studies where cardiomyocyte cell strands had been subjected to 10.5% global static strain and where conduction was assessed using voltage sensitive dyes (ATD: +3.2% vs. +3.3%; *θ* ː +7.1 vs. +7.0; maximal action potential upstroke velocities and *t*_DS_ not affected by strain)^33^. The overall concordance of results obtained with the two different measurement techniques validates the MaMEA and demonstrates that measurements over 2.2 mm long strained regions (as opposed to 5 mm in the former study) are sufficient to obtain consistent results. Going beyond these findings, the present study showed that ATDs scale linearly with strain within the range of strain amplitudes tested. When calculating *θ* based on the actual lengths of the strained preparations divided by the respective ATDs, the resulting *θ* vs. strain relationship showed a $\frac{\theta }{{\theta _0}} = \frac{{\varepsilon + 1}}{{\alpha \varepsilon + 1}}$ dependence. Based on cable theory and as further described in the Supplementary Note 10, the relationship can be explained by strain-dependent changes of axial resistance under the assumption that cytoplasmic resistance contributes 29% and gap junctional resistance 71% to total axial resistance which is in accordance with recently published values obtained with the same type of preparation (25%:75%)^3^. Beyond these results, the MaMEA experiments demonstrated conclusively that conduction in strands of cardiomyocytes is insensitive to strain rates exceeding physiological values by an order of magnitude (18 s^−1^ at 10% strain amplitude). This surprising observation made in a total of six independent preparations (see Supplementary Note 11) suggests that cardiomyocytes can endure substantial mechanical stress without undergoing measurable electrophysiological changes at the cellular and multicellular level. The question then remains as to which mechanism(s) are responsible for heavy impact induced cardiac arrhythmias. Apart from the caveats (i) that the findings presented were obtained in vitro where MEC mechanisms may differ from those present in vivo, (ii) that conduction was determined 300 ms after abruptly increasing the strain, and (iii) that maximal strain amplitudes were possibly too moderate, the findings suggest that cells different from cardiomyocytes may be responsible for strain-dependent induction of cardiac arrhythmias with electronically coupled cardiac myofibroblasts being possible candidates^33^.

Given that the MaMEA is potentially able to generate strain rates reaching 1000 s^−1^ ^10^ while simultaneously providing correlated strain amplitude modulation and electrophysiological recordings, the technology presented is ideally suited to systematically address fundamental questions regarding cellular mechanisms underlying MEC in the context of very fast strain events in excitable tissues.

## Methods

### Device fabrication

Sylgard 186 (Dow Corning) was casted as a sheet over a sacrificial polyacrylic acid layer. Target thickness was 80–125µm. The sheet was mounted on a circular frame with a bi-axial pre-stretch of 1.2 × 2.7, resulting in a 25-µm-thick membrane as measured by optical interferometry. The addition of electrodes and the assembly of the culture wells is illustrated in the Supplementary Fig. 2. Carbon powder (Ketjenblack EC300; AkzoNobel) embedded in an elastomeric matrix was pad printed on both sides of the free-standing membrane. A ~2 µm Silbione liquid silicone rubber (LSR 4305; Bluestar) isolation layer was pad printed to electrically insulate the strain electrodes from the culture medium and cells. The recording electrodes, 200 µm wide, were gold ion implanted with 2.5 keV^11^, and patterned using a steel shadow mask. A subsequent LSR 4305 layer was stamped to electrically insulate the Rec_Els_ from the bath. Openings were included for cell contact. The patterned membranes were transferred to the PCB frames, electrical contact between the PDMS membrane and the PCB pads was ensured using SS-25 EMI/RFI conductive adhesive (Silicone Solutions). The DC resistance of the Rec_Els_ channels ranged from 300Ω to 1.5kΩ and their AC resistance (impedance) measured at 1 kHz through the bath ranged from 1.7 to 9.6kΩ. DC and AC impedances changed <6% for an applied strain of 10% in *Z*_*ε*+_. Finally, the culture well was added to the device and sealed using LSR 4305. The cells were patterned using a Mylar mask. A Mylar backing immobilized the suspended membrane prior to the strain experiments and was left in place for the control experiments. Images of the electrodes and the cell strand are presented in Supplementary Figs. 5 and 6.

### Device characterization

The DEA voltage–strain relation was measured using  widefield microscopy. For calibration purposes, the devices were incubated with cell culture medium for ≥4 days. Image processing (National Instruments vision assistant) was used for edge detection and strain calibration. A high-speed camera served to capture the strain response. The strain–voltage relation was tracked at 10 Hz frame rate while ramping the voltage linearly from 0 to 3.6 kV over 30 s (Fig. 2a). The dynamic response was tracked at a frame rate of 1 kHz. The five ramp times were recorded over a total of 300 ms (Fig. 2b). The derivative of these data reflects the imposed strain rate. The corresponding voltage ramps are illustrated in Supplementary Fig. 4. The strain during the standard protocol (Fig. 2c) was calibrated optically at 100 Hz frame rate for each step function at multiple drive voltages.

### Cell culture

Primary cultures of neonatal rat ventricular cardiomyocytes were obtained using established procedures and following Swiss guidelines for animal experimentation under the licencse BE27/17 of the state veterinary department of the canton Bern^34^. In brief, hearts from eight to ten neonatal rats (Wistar, 1-day-old) were excised, the ventricles were minced with scissors and the resulting small tissue pieces dissociated in Hank’s balanced salt solution without Ca^2+^ and Mg^2+^ (BioConcept, 3–02F29-I) containing trypsin (0.1%; Sigma, T4674) and pancreatin (120 µg/ml; Sigma, P3292). The dissociated cells were, after centrifugation, resuspended in medium M199 with Hank’s salts (Sigma, M7653) supplemented with penicillin (20 U/ml; Sigma, P7794), vitamin B_12_ (2 µg/ml; Sigma, V2876), bromodexyuridine (100 µmol/L; Sigma, B-5002), vitamin C (18 µmol/L; Sigma, A-4544), epinephrine (10 µmol/L; Sigma, E4256), l-glutamine (680 µM/L; Sigma, G7513) and 10% neonatal calf serum (Biochrom, Bioswisstec, S0125). The cell suspension underwent differential preplating in cell culture flasks for 120 min. The supernatant containing predominantly cardiomyocytes was collected, cell densities determined by with a hemocytometer, and cell density adjusted by dilution as to result in a seeding density of 3500 cardiomyocytes/mm^2^. Before seeding, the MaMEA membrane was activated by ultaviolet-light exposure (8 min, UVO-Cleaner Model No. 342, Jelight Company Inc.) and subsequently coated with collagen type IV (C5533, Sigma). Patterned cell growth was realized using a lift-off technique. For this purpose, laser cut Mylar films that carried the negative of the desired cell pattern were affixed to the membrane prior to cell seeding. The cultures were kept in an incubator at 36 °C in a humidified atmosphere containing 0.5% CO_2_. Medium was exchanged 24 h post seeding with supplemented medium M199 (5% serum) and every other day thereafter. Mylar masks were peeled off at the time of the first medium exchange resulting in well-defined strands of cardiomyocytes (see Supplementary Note 5). Experiments were performed 72–96 h after seeding of the cardiomyocytes. hi-PSC-derived cardiomyocytes were obtained from Ncardia (Pluricyte^®^ Cardiomyocyte Kit, Ncardia, Germany). Strand preparations were generated using the patterning procedures described for the rat cardiomyocytes above. Handling and culturing of the stem-cell-derived cardiomyocytes was done according to the detailed instructions given by the manufacturer. Experiments were performed with 4-day-old preparations.

### Experiments

All experiments were performed in an incubator set to 36 °C/0.5% CO_2_. During experiments lasting ≤3 h, the MaMEA wells were covered by a lid. The MaMEA was mounted to the motherboard that was plugged into a DAQ (6 µV resolution, 4 mV dynamic range) residing in the incubator. After assembly in the incubator, the system was left to stabilize for 30–60 min. Experiments were started once the drift in ATDs became negligible. To be included in experiments, the following criteria had to be met by the preparations: (i) ≥5 recording electrodes were operational, (ii) the DEA actuator was able to generate ≥10% strain without breakdown, and (iii) cell strands displayed a uniform structure and displayed continuous conduction under control conditions.

### High-voltage drive and data recording

The high-voltage signal (*V*_*ε*_) was generated by an arbitrary function generator (Agilent 33521A) and amplified by a factor of 500x using an HVA series amplifier (Ultravolt) with a bi-polar output of max 5 kV and a slew rate of 40 Vµs^−1^, resulting in a theoretical minimum charge time of 0.1 ms (no load). The signal was filtered by a low-pass filter (LPF, 1 kHz cutoff, resulting in a minimum 5 ms rise time). The applied voltage was monitored using an oscilloscope. In addition to step functions, the DEA voltage was also ramped as ~$\root {3} \of {{\frac{t}{{t_0}}}}$, resulting in a linear strain ramp exhibiting a pre-determined ramp rate. Only the standard protocol strain profiles were imposed on the cell strand during measurements.

### Electrical and mechanical stimulation protocol

Preparations were stimulated at double threshold amplitude with a biphasic current pulse generated by a custom-made stimulator. For each data set, the cell strand was stimulated for 30 s (20 s pre-stimulation, 10 s recorded) before switching on the DEA voltage (100 ms after the last activation of the preparation) imposing tensile and compressive strain on the preparation. The standard protocol was based on 10 strain cycles at 4.8 s period and a 50% duty cycle. The amplitude of the DEA voltage ranged from 1 to 3.6 kV and the ramp time was set to 5, 20, 50, 100, or 200 ms. For each data set, the slowest and smallest amplitude was tested first (200 ms rise time to 0.9 kV) followed by increasing the amplitude at constant ramp times. The data set was concluded with the 5 ms ramp sequence to 3.6 kV that was followed by a 10-s-long recording of stimulated AP_ECs_ under non-strained conditions. The overall measurement protocol lasted 10 + 48 + 10 s = 68 s during which each electrode recorded 170 AP_ECs_.

In all experiments, an initial 68 s of baseline recording was performed (electrical but no mechanical stimulation) that was used to characterize basic electrophysiological properties of the strand. Performance parameters and stability are illustrated in Supplementary Figs. 8 and 9.

### AP_EC_ analysis

Electrograms were analyzed using MATLAB following the procedures described in Supplementary Note 6. Data were blanked, that is, set to background level, during the electrical stimulation artifacts. Similarly, during the *V*_*ε*_ ramp causing the mechanical stimulation, capacitive coupling resulting in electrical noise prohibiting recording of APs and, consequently, the period of DEA modulation was blanked as well. The shortest blanking could be as brief as 10 ms, but always needed to exceed the ramp time. Given that the first AP was electrically triggered 300 ms after the mechanical stimulation, the *V*_*ε*_ blanking did not interfere with the recording of electrograms (for details see Supplementary Fig. 7).

Detailed procedures regarding the data evaluation protocol are described in Supplementary Note 6. Electrical noise levels are illustrated in Supplementary Fig. 7, and an example of stability and timing accuracy during a baseline recoding is presented in Supplementary Fig. 8.

A control experiment was performed to determine the effect of the DEA voltage in the absence of mechanical strain. For this purpose, the membrane was immobilized by leaving the Mylar on the substrate while actuating the DEA up to 3.5 kV. Results are depicted in Supplementary Fig. 14 (Supplementary Note 12), with the corresponding *p* value indicating that control values were not different from immobilized values (*p* = 0.31) while, as expected, ATD changed significantly for both strain zones after removal of Mylar. This corroborates previous findings with DEA actuators where, in the presence of an immobilized membrane, electrical fields had no measurable effect on cell cultures^15^.

To exclude that HV, capacitively coupled to the cultured cells, could cause adverse effects on cell viability, cardiomyocytes were grown on an array of metallic electrodes covered with 100 µm of PDMS and were exposed to a modulating high-voltage signal of 4 kV at 1 Hz over 6 h. A cell viability kit (Biotium #30065) revealed no differences in the density of apoptotic and necrotic cells when compared to control samples (the data are presented in the Supplementary Fig. 16, a discussion on this topic can be found in Supplementary Note 13, including a graphical illustration of electric field strengths found in biological systems presented in Supplementary Fig. 15).

### Ethical compliance

The experiments are compliant and were performed following strict federal regulations under license BE27/17 of the State Veterinary Department of the Canton of Bern.

### Reporting summary

Further information on experimental design is available in the Nature Research Reporting Summary linked to this article.

## Supplementary information

Supplementary Information

Description of Additional Supplementary Files

Supplementary Movie 1

Reporting Summary

## Data Availability

Source data underlying Fig. 5 and Supplementary Fig. 13 are provided as a Source Data file (10.5281/zenodo.2541654). All other relevant data are available from the authors.
