# Supplementary material for: High-speed mechano-active multielectrode array for investigating rapid stretch effects on cardiac tissue
Source: Nat Commun. 2019 Feb 19;10:834. doi: 10.1038/s41467-019-08757-2 (PMC6381132; doi:10.1038/s41467-019-08757-2)
Supplement: Supplementary file 1 — Supplementary Information [file 41467_2019_8757_MOESM1_ESM.pdf]

Supplementary Material

High-speed Mechano-active MEA for Investigating Rapid Stretch Effects on Cardiac Tissue

Matthias Imboden, Etienne de Coulon, Alexandre Poulin, Christian Dellenbach, Samuel Rosset, Herbert Shea, Stephan Rohr

Table of Contents

Supplementary Note 1:	Dielectric Elastomer Actuators: Principles of Operation	3
Supplementary Note 2:	Device Fabrication and Preparation	4
Supplementary Note 3:	Multi-MaMEA System with Signal Conditioning	6
Supplementary Note 4:	Characterization of Mechanical Strain in the Voltage and Time Domain	7
Supplementary Note 5:	Patterning Cardiomyocyte Strands & Electrical Stimulation	9
Supplementary Note 6:	Detection and Analysis of Extracellular Action Potentials (AP_{EC}s)	10
Supplementary Note 7:	Stability of Recordings with Ion Implanted Electrodes	12
Supplementary Note 8:	Detection of Action Potential Repolarization in Strands of Neonatal Rat Ventricular Cardiomyocytes and hi-PSC Derived Cardiomyocytes	16
Supplementary Note 9:	Deriving θ from Activation Time Differences	18
Supplementary Note 10:	Cable Equation Applied to a Linearly Strained Cell Strand	19
Supplementary Note 11:	Effects of Strain on Impulse Conduction in Cardiomyocyte Cell Strands - Additional Experiments	21
Supplementary Note 12:	Control Experiments with Mechanically Immobilized PDMS Membranes	23
Supplementary Note 13:	Effects of High Electrical Field Strengths on Cardiomyocytes	25
References		27

Abbreviations

PDMS	polydimethylsiloxane
MaMEA	mechano-active multielectrode array
PCB	printed circuit board
DEA	dielectric elastomer actuator
DEA_{El}	DEA electrode
Rec_{El}	extracellular recording electrodes
$Stim_{El}$	stimulation electrode
ECP	extracellular potential
AP_{EC}	extracellular action potential
DAQ	digital control and acquisition system
ESD	electrostatic discharge

t_{AT}	local activation time, index defines the recording electrode
t_{ramp}	time of voltage ramp to change the strain state
ATD	activation time difference, indices define the recording electrodes
BCL	basic cycle length
V_{ECP}	extracellular potential, index defines the recording electrode
V_{pp}	peak to peak AP_{EC} amplitude, index defines the recording electrode
t_{DS}	AP_{EC} downstroke time, index defines the recording electrode
dV_{ECP}/dt_{max}	maximal AP_{EC} downstroke velocity
I_{stim}	bi-polar stimulation current amplitude
θ	impulse propagation velocity, index defines the zone
$Z_{\varepsilon+}$, $Z_{\varepsilon-}$	positive and negative strain zones
ε_{+}	strain in zone $Z_{\varepsilon+}$ (or $Z_{\varepsilon-}$)
$\lambda_{\varepsilon}=1+\varepsilon$:	stretch
V_{ε}	actuation potential across the DEA

Supplementary Note 1: Dielectric Elastomer Actuators: Principles of Operation

Dielectric elastomer actuators (DEAs) consist of stretchable electrodes that are used to generate an electrostatic pressure that acts on a dielectric membrane consisting of polydimethylsiloxane (PDMS). As the PDMS is deformable and at the same time incompressible (Poisson's ratio $\nu = 0.5$), the attraction of the DEA electrodes causes a lateral deformation which results in a strain field.[1] To enable the DEA electrodes to follow expansion during operation, they are implemented by using carbon powder bound to the PDMS[2]. Supplementary Fig. 1a illustrates the actuation principles and Supplementary Fig. 1b illustrates the implementation of a DEA as used in this study. The device shown exhibited a large strain response (19% at 4 kV). After seeding of the cells, the strain response is reduced to ~ 10-12% at 4 kV which is likely due to stiffening of the membrane following UV activation that is required for cell attachment (cf. method section).

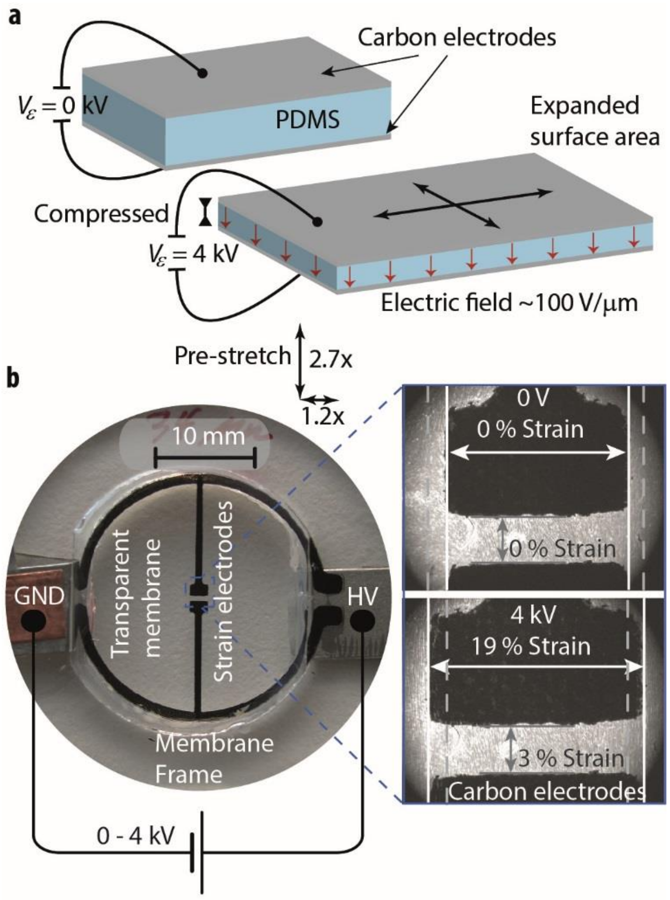

Supplementary Figure 1. Dielectric Elastomer Actuators (DEAs). **a)** Working principle with the elastomer sandwiched between compliant carbon electrodes being compressed during application of high voltage. **b)** General layout of our uniaxial strain actuator with illustration of deformation during actuation.

Prior to mounting to the holder, the PDMS membrane is pre-strained for 2 reasons: 1) It has been shown that pre-strain improves the performance by delaying mechanical buckling[2] and by increasing the electrical breakdown field. 2) Pre-strain is mandatory to obtain uniaxial strain during DEA actuation: whereas, without pre-strain, expansion would be uniform in all directions (as shown in panel a)), the hyperplastic properties of PDMS in presence of different pre-stretches along the orthogonal axes permits immobilization of one axis[3,4] as shown in panel b. It has been demonstrated that pre-stretch amounting to 2.7-fold (*y*-direction)

and 1.2-fold (x-direction) sufficiently stiffens the membrane along one axis[5] as to produce linear strain along the low pre-stretch axis only.

The stamping method[6] allows the transfer of patterned electrodes onto the suspended and pre-strained membrane (see Supplementary Note 2 for details on the fabrication steps and materials used). The geometry of the DEA electrodes is chosen such that the center of the membrane remains transparent for imaging purposes. This central region experiences uniform uniaxial tensile strain ($Z_{\varepsilon+}$) during DEA actuation. With a total well diameter of 20 mm, the expansion of the membrane between the center DAE electrodes over a distance of ~ 2 mm is compensated for by compressive strain in the ~8 times larger outer regions ($Z_{\varepsilon-}$) which results in a correspondingly lower compressive strain in the $Z_{\varepsilon-}$ zones. Typical data describing strain field homogeneity and residual strain perpendicular to the main axis are presented in the supplementary material of Ref.[5] Of note, it has been shown previously that strain perpendicular to the direction of impulse conduction does not alter ATDs.[7]

Supplementary Note 2: Device Fabrication and Preparation

The fabrication steps of the mechano-active multielectrode array (MaMEA) are summarized in Supplementary Fig. 2 with further details regarding DEA fabrication given elsewhere.[5,6,8] Membranes of Sylgard 186 (Dow Corning) are cast to the desired thickness (125 µm) and then pre-stretched 2.7-fold (y-direction) and 1.2-fold (x-direction) before being fastened to circular holders with ARClear pressure activated double sided adhesive (Adhesive Research). The suspended membranes are typically 24 µm - 32 µm thick as determined by optical interferometry.

The DEA electrodes consist of a mixture of carbon black powder (Ketjenblack EC300; AkzoNobel) and Silbione liquid silicone rubber (LSR 4305; Bluestar) (see refs[2,6] for details). The pattern is transferred to both sides of the PDMS membrane using pad printing techniques. Resolution and alignment accuracy of this process is on the order of 100 µm. (Supplementary Fig. 2a,b). After curing the carbon electrodes at 80°C for 60 minutes in an oven, they are passivated by stamping ~2 µm of LSR 4305 on top of the electrodes. This insulates the high voltage electrodes both in respect to the culture medium (top) and the air (bottom) which is critical for improving the lifetime and reliability of the device. Moreover, the bottom passivation reduces electrical noise that may arise from micro-burst of currents secondary to the ionization of air.

Extracellular recording and stimulation electrodes (Rec_{El} and $Stim_{El}$) are fabricated using gold ion implantation techniques and steel shadow masks of the desired shapes (Supplementary Fig. 2c).[9] At the site of prospective contacts with the biological preparation, recording electrodes are 200 μm wide. Electrodes are passivated using stamping techniques with the exception of the tip of $Stim_{Els}$ and the center region of Rec_{Els}.

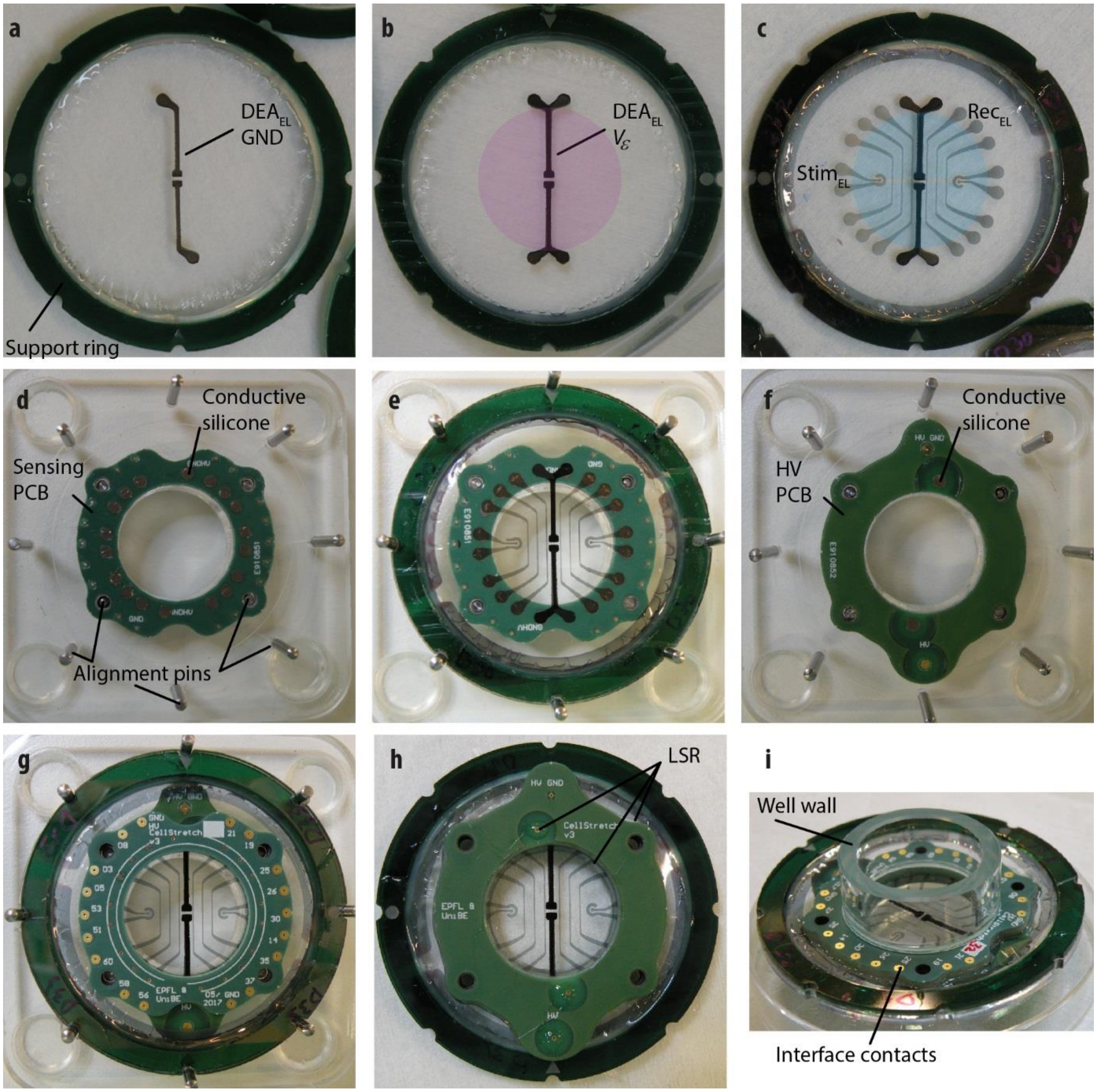

Supplementary Figure 2. Fabrication steps after the PDMS membrane is stretched and mounted to the carrier ring. **a)** Carbon electrodes stamped on front side (ground electrode) and, **b),** on backside (high voltage electrode). Both sides of the membrane are subsequently passivated by stamping, with the exception of the contacts (pink shading). **c)** Ion gold implanted stimulation and recording electrodes. Electrodes are passivated (blue shading) except for the end ($Stim_{El}$) and center (Rec_{El}) which will establish contact to the excitable tissue. **d)-h)** Transfer of the PDMS membrane to the PCB frames with electrical contact being made with carbon containing silicone. **h)-i)** The device is sealed using LSR and the PMMA wall forming the culture well is added.

The transfer of the fully structured membrane to the PCB frames and the assembly of the cell culture well is illustrated in Supplementary Fig. 2d-i. ARClear is used to mount the membrane to the PCB. Furthermore, the adhesive is used as a mask for the SS-25 EMI/RFI conductive adhesive (Silicone Solutions) that electrically connects the PCB pads to the carbon and gold electrodes on the membrane. First, the sensing (top) PCB is treated with ARClear and SS-25 EMI/RFI and the membrane is mounted to the PCB (Supplementary Fig. 2d,e). Subsequently, the same procedure is repeated for the high-voltage bottom PCB (Supplementary Fig. 2f,g), thereby sandwiching the membrane between the two PCBs.

LSR 4305 is used to seal the interface between the PDMS membrane and the PCBs. This prevents the occurrence of leaks and improves device durability and reliability by ensuring a gradual transition between the free-standing elastic membrane and the solid PCBs. Exposed HV-vias are also sealed by RTV (room temperature vulcanized silicone rubber, Silpuran 4200; Wacker Chemie AG) to prevent electrical break-down (cf. Supplementary Fig. 2h). The device is completed by fixing the wall of the cell culture well (poly-methyl methacrylate, PMMA) to the device with LSR 4305.

Prior to the cell seeding, the PDMS membrane is immobilized by adding a sheet of Mylar to the backside. Immobilization has proven to be critical for optimal cell adherence possibly by removing vibrations of the thin membrane. A second Mylar mask carrying the desired growth pattern is added to the top of the membrane. At this point, the MaMEA is complete and ready for cell seeding as described in the methods section. It has been observed that cell growth is improved if the wells are placed in the incubator for ~ 48 hours prior to cell seeding (without growth medium). The exact reason for this is not known but may involve saturation of the PDMS membrane with water in the humid atmosphere.

Supplementary Note 3: Multi-MaMEA System with Signal Conditioning

Because the MaMEA architecture lends itself for conducting multiple experiments in parallel, we designed the multi-MaMEA dish shown in Supplementary Fig. 3. In contrast to the single well MaMEAs, the multiwell configuration included signal conditioning circuits for each well for buffering and low gain amplification (3.5x) of the extracellular signals. On-board signal conditioning close to the Rec_{Els} reduces the requirements on the DAQ and reduces noise pickup. Signal conditioning close to the recording sites also minimizes the capacitive load which can affect AP_{EC} amplitudes. Accordingly, the signals shown are

qualitatively at least similar to the ones recorded in single well devices as used for the experiments shown in the main manuscript. Conceptually more important is the ability to run experiments in parallel, which improves throughput and allows for more comprehensive studies and/or redundancies. Unlike cell stretchers based on pneumatics or conventional motors for actuation, the MaMEA system is intrinsically parallelizable in the sense that a single high voltage source and appropriate transistors can be used to generate a whole range of drive signals which can then be applied individually to each well because each well has a dedicated HV input for strain actuation.

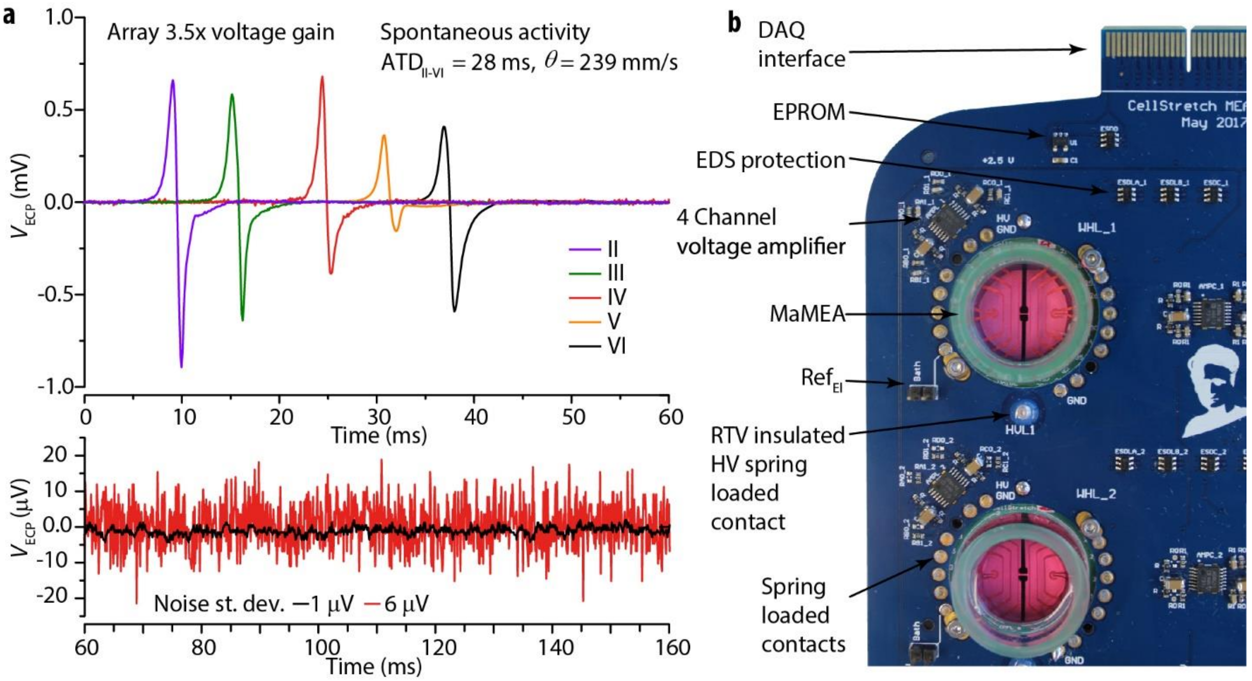

Supplementary Figure 3. Multi-MaMEA system. **a)** AP_{EC} recordings with buffer amplifier (gain 3.5x) with improved noise levels. Bottom panel shows the maximum and minimum noise observed in this MaMEA. **b)** Close-up view of the Multi-MaMEA board containing a total 6 individually addressable MaMEAs with dedicated onboard amplifiers.

Supplementary Note 4: Characterization of Mechanical Strain in the Voltage and Time Domain

To determine the extent and dynamics of strain imposed during experiments, the devices need to be calibrated under experimental conditions, i.e., the MaMEA must be held at physiological temperature and be filled with growth medium. Strain profiles in response to specific actuation voltage profiles as measured by a high-speed camera under these conditions are depicted in Supplementary Fig. 3. Step increases in voltage result in an approximately exponential mechanical response with dynamics being limited by the slew

rate (40 V/µs) and compliance (I_{max} = 200 µA) of the HV source (HVA ±5 kV precision high voltage amplifier; Advanced Energy) and by the 1 kHz low pass filter (LPF) inserted between the HV amplifier and the MaMEA. In presence of the LPF, we observe a voltage rise time to 95 % of the full amplitude of 4.7 ms which defines the minimum t_{ramp}. Once maximal voltage is reached, strain continues to rise slightly over timescales in the order of seconds as the membrane adapts to the compressive strain and the new electrical boundary condition (mechanical creep). As explained in the manuscript, linear strain ramps are obtained by increasing the DEA actuation voltage as $V_\varepsilon = V_{\max} \times \sqrt[3]{\frac{t}{t_{\text{ramp}}}}$. Supplementary Fig. 4 shows the time course of strain development for t_{ramp} values amounting to 5 ms, 20 ms, 100 ms and 200 ms which result in strain rates ranging from 0.5 /s to almost 20 /s.

For the experiments described in the main manuscript, individual snapshots of the preparations were taken during the measurement protocols using a USB microscope (Dino-Lite Premier AM4113T) which served as calibration images. Post experiments, precise strain profiles were recorded with a high-speed camera (Eosens MC1362; Mikrotron). The camera was operated at 1000 fps to characterize the strain ramps and at 100 fps to measure the strain profile during the entire experimental protocol. Precise strain amplitudes were obtained after recalibration of the high-speed images based on the calibration images obtained during the actual experiments. While errors of strain determinations based on image analysis alone may be as low as 0.01% (measurement noise), we estimate conservatively that rescaling procedures and variations between measurements permitted to reach an accuracy better than 5% of a given strain amplitude (*i.e.* 0.5% at 10% strain).

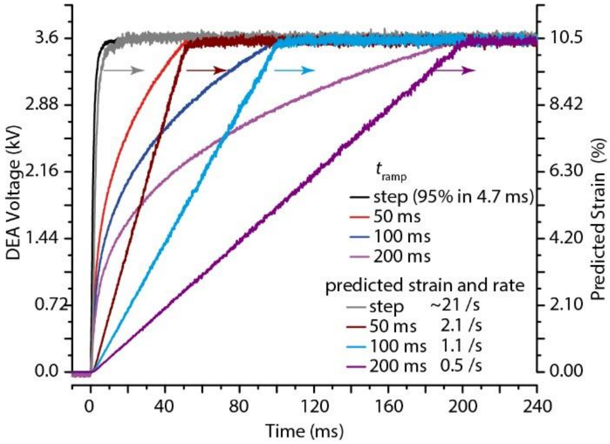

Supplementary Figure 4. DEA actuation voltage and strain development as a function of time. The axis on the right denotes strain predicted by the 3rd root voltage actuation profile. Data are shown for the ramp times 5 ms (step), 50 ms, 100 ms and 200 ms. They illustrate that the voltage actuation profiles result in linear strain ramps (constant strain rate).

A manuscript detailing the electro-mechanical properties of the device, including alternate actuation geometries enabling stretch and compression, is in preparation.[10] That study focuses, in part, on maximizing strain rates based on feedforward drive techniques where strain rates close to 1000 /s can be achieved. Such rates exceed

those offered by motor or hydraulically driven cell stretchers by three orders of magnitude. Further details on the mechanical performance of similar devices can be found in ref [10] and in the supplementary information of ref [5].

Supplementary Note 5: Patterning Cardiomyocyte Strands & Electrical Stimulation

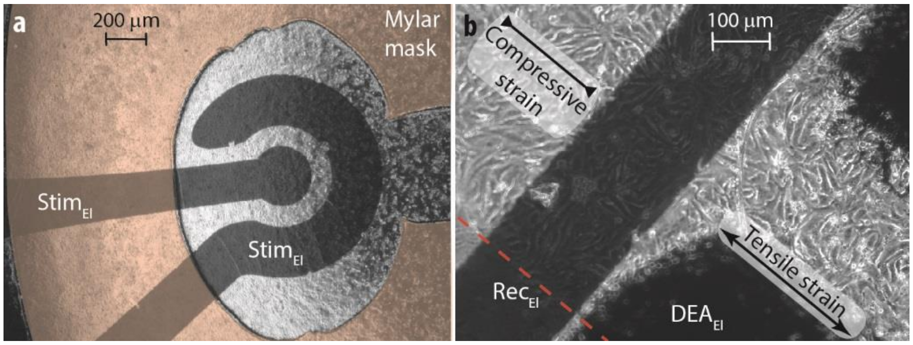

Supplementary Figure 5. Phase contrast images of cardiomyocytes grown on PDMS membranes. **a)** Region containing the electrical stimulation electrode (Stim$_{El}$) with the cell growth patterning mask (Mylar) still in place (brown). **b)** Electrical sensing electrode (Rec$_{El}$) and mechanical actuation electrode (DEA$_{El}$) being traversed by a 2 mm wide cardiomyocyte strand (border indicated with red dashed line).

Microscopic images of cardiomyocytes grown in the MaMEA are depicted in Supplementary Fig. 5. Supplementary Fig. 5a shows the dense layer of cardiomyocytes in the region of the electrical stimulation electrodes (Stim$_{El}$) before removal of the Mylar mask that serves to define the growth pattern. Supplementary Fig. 5b depicts the cell strand passing over a recording electrode (Rec$_{El}$) and through the central region between the DEA electrodes (DEA$_{El}$). Out of plane motion of the strained region during DEA actuation is minimal, allowing for real-time imaging of the cells as they are being stretched (cf. Supplemental movie 1). Thus, in addition to electrical measurements, optical assessments of cells including fluorescence imaging can be performed during stretch.[11]

Supplementary Fig. 6 shows an overview of the MaMEA substrate with the cardiomyocyte cell strand being outlined by a green trace. The strand runs between the outer stimulation electrodes, over a total of six electrical sensing electrodes, and through the central region undergoing uniaxial homogeneous tensile strain upon DEA actuation. While the top carbon electrodes shield the cells from the HV electrodes on the backside of the PDMS membrane, fringe fields and slight inaccuracies in electrode alignment possibly result in significant exposure of cardiomyocytes to large electric fields. This issue is addressed in Supplementary Notes 12 and 13.

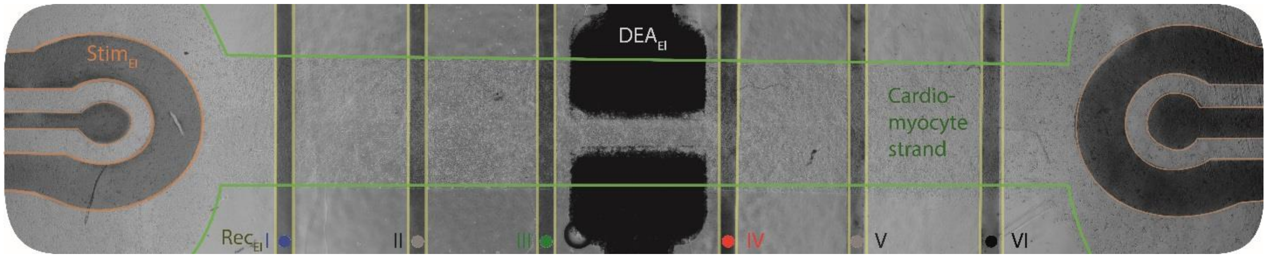

Supplementary Figure 6. Overview of the bottom of a MaMEA device with the cardiomyocyte strand covering the stimulation electrodes, Stim$_{Els}$, recording electrodes, Rec$_{Els}$ and the central strain electrodes, DEA$_{Els}$.

Preparations were stimulated by a bi-polar current pulse provided by a current source that was galvanically insulated from the bath/sensing circuit to reduce electrical crosstalk with the recording electrodes. Following the determination of minimal current amplitudes necessary for successful stimulation at a stimulus duration of 2-4 ms, preparations were stimulated at twice this value (typically 20-60 µA). For some experiments, preparations were excited just above threshold to minimize electrical crosstalk with the recording electrodes.

Supplementary Note 6: Detection and Analysis of Extracellular Action Potentials (AP$_{ECs}$)

Supplementary Fig. 7a depicts the extracellular potential (V_{ECP}) measured with a Rec$_{El}$ over a 500 ms period during which the preparation is stimulated twice with V_ε being ramped to 3.6 kV during 5 ms between the two stimulated APs. The red trace depicts the raw data recorded by the DAQ at 10 kHz sampling rate. The peak-to-peak noise of the recording is <10 µV which corresponds to a spectral noise density of 0.1 $\mu\mathrm{V}/\sqrt{\mathrm{Hz}}$. The standard deviation of the noise is 2 µV, as can be seen in the close-up plot of the first 300 ms of the recording in Supplementary Fig. 7b. In order to detect and characterize AP$_{ECs}$ during propagated activity, the following analysis steps were performed (Matlab):

1. Artifacts due to electrical crosstalk with stimulation and DEA electrodes were blanked (data set to 0). Typical blanking periods to remove the I_{stim} artifact lasted 5 ms, blanking for removing the V_ε artifact were chosen to be slightly longer than the ramp duration.
2. The derivative was calculated and the output smoothed (Savitzky-Golay filter, with n = 5 to 20 depending on noise levels; smoothing with n>5 tended to have a significant impact on the measured amplitude but had negligible impact on the timing because the derivative is symmetric around t_{AT}). The filtered result is shown by the purple trace in the lower panel of Supplementary Fig. 7.

3. From the derivative, AP_{ECs} were identified using peak detect functions (Matlab function findpeaks())and thresholds tuned to each Rec_{EI} for a given experiment. Filters were applied to remove noise spikes.
4. Once a peak was detected, its "energy" was measured by summing up the squares of its amplitude over the downstroke period of the AP_{EC} as defined by the negative derivative. This provided a second threshold for peak selection and was effective in excluding noise spikes which may be large but are shorter than AP_{ECs}.
5. Finally, "true" AP_{ECs} were selected based on the plausibility argument that AP_{ECs} must occur within a limited time period after electrical stimulation. This time varies with θ and with the distance between the $Stim_{EI}$ and a given Rec_{EI} and lies within 8-100 ms of the stimulation trigger.
6. Each identified peak was fitted to a Gaussian (sup. eqn. (S1)) to determine the activation time, t_{AT}, the downstroke time, t_{DS}, and the maximum downstroke amplitude, $\left|\frac{dV_{\mathrm{ECP}}}{dt}\right|_{\max}$. The data points within ± 0.4 ms of the peak were included in the fit (9 data points).
7. Activation time, t_{DS}, and maximal downstroke velocity of the AP_{EC} were determined from Gaussian fit to the derivative of the downstroke of the signal as follows:

Consider the Gaussian expressed as

$$\frac{dV_{\mathrm{ECP}}}{dt} = y_0 + Ae^{-\frac{1}{2}\left(\frac{t-t_0}{w}\right)^2}, \tag{S1}$$

where y_0 is an offset, A is the amplitude of the Gaussian curve, t_0 the center, and w characterizes the width.

From this the activation time (t_{AT}), the downstroke time (t_{DS}), and maximal downstroke velocity ($\left|\frac{dV_{\mathrm{ECP}}}{dt}\right|_{\max}$) (defined through the zero crossings) were extracted as follows:

$$t_{\mathrm{AT}} = t_0, \tag{S2}$$

$$t_{\mathrm{DS}} = 2\,w\sqrt{2\ln\left(-\frac{A}{y_0}\right)}, \tag{S3}$$

$$\left|\frac{dV_{\mathrm{ECP}}}{dt}\right|_{\max} = |y_0 + A|, \tag{S4}$$

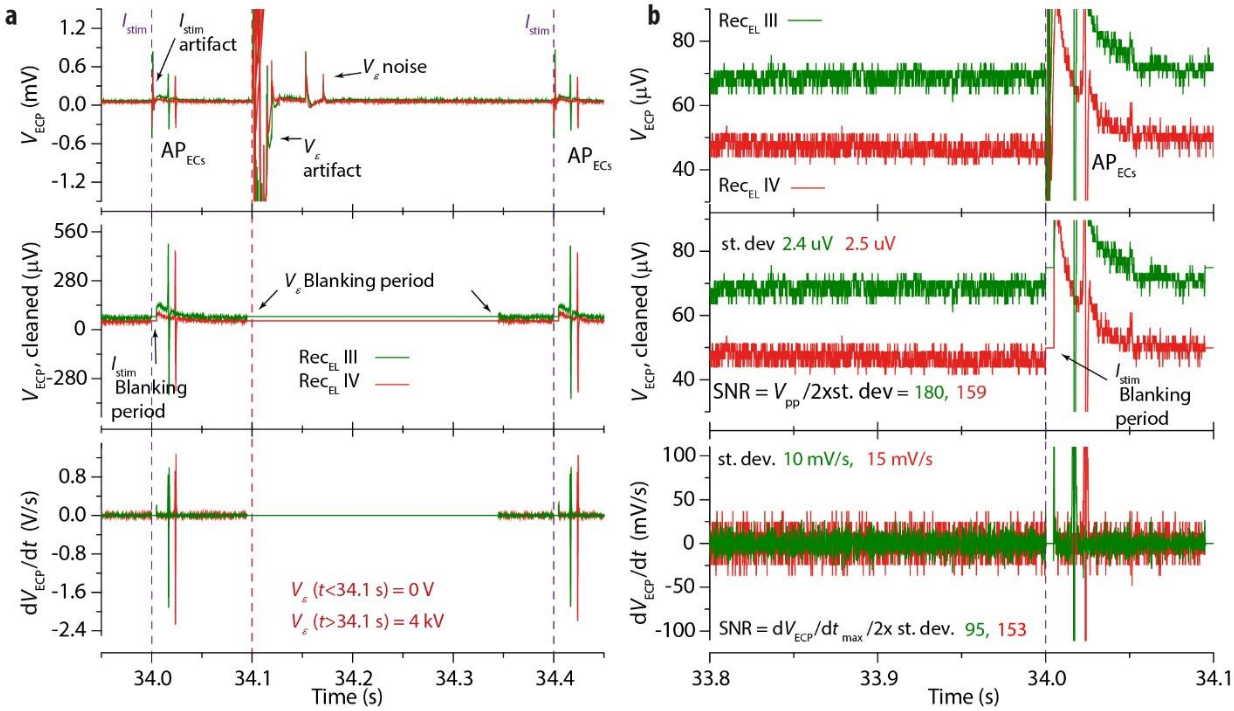

Supplementary Figure 7. V_{ECP} and noise characterization. **a)** Two sequential AP_{ECs} recorded by two electrodes (Rec_{El}III and Rec_{El}IV straddling the tensile strain zone) before and after the strain voltage is turned on. Top panel: raw data recorded at 10 kHz sampling rate with a 20 Hz LPF show, besides the AP_{ECs}, the stimulation and DEA actuation artifacts. Middle panel: data after blanking of the artifacts. Lower panel: derivative of the V_{ECP} data after smoothing. **b)** Magnified views illustrating the noise level in the signals in the vicinity of the first stimulated AP_{EC}. The standard deviation is on the same order as the discretization of the data and the signal-to-noise level (peak-to-peak) in this particular case amounts to ~ 100.

Supplementary Note 7: Stability of Recordings with Ion Implanted Electrodes

Comparison of strain-related changes in AP_{EC} characteristics and impulse conduction during experiments lasting > 1 minute are critically dependent on stable baseline conditions in regard to θ and AP_{EC} characteristics. A zero strain characterization of ATDs, θ, V_{pp}, t_{DS} and $\left|\frac{dV_{\mathrm{ECP}}}{dt}\right|_{\max}$ as monitored by four Rec_{Els} over 68 s in a preparation paced at 150 BPM is presented in Supplementary Fig. 8. These measurements were obtained from the same culture as presented in the manuscript and were recorded after all strain modulation protocols and hence illustrate the stability after prolonged mechanical perturbations. V_{pp} is measured from the raw data by subtracting the minimum from the maximum value within ±3 ms of the zero crossing of the AP_{EC} signal. All other parameters are determined as described above. In addition to the absolute values recorded by each of the four electrodes, relative values for the positive strain region and

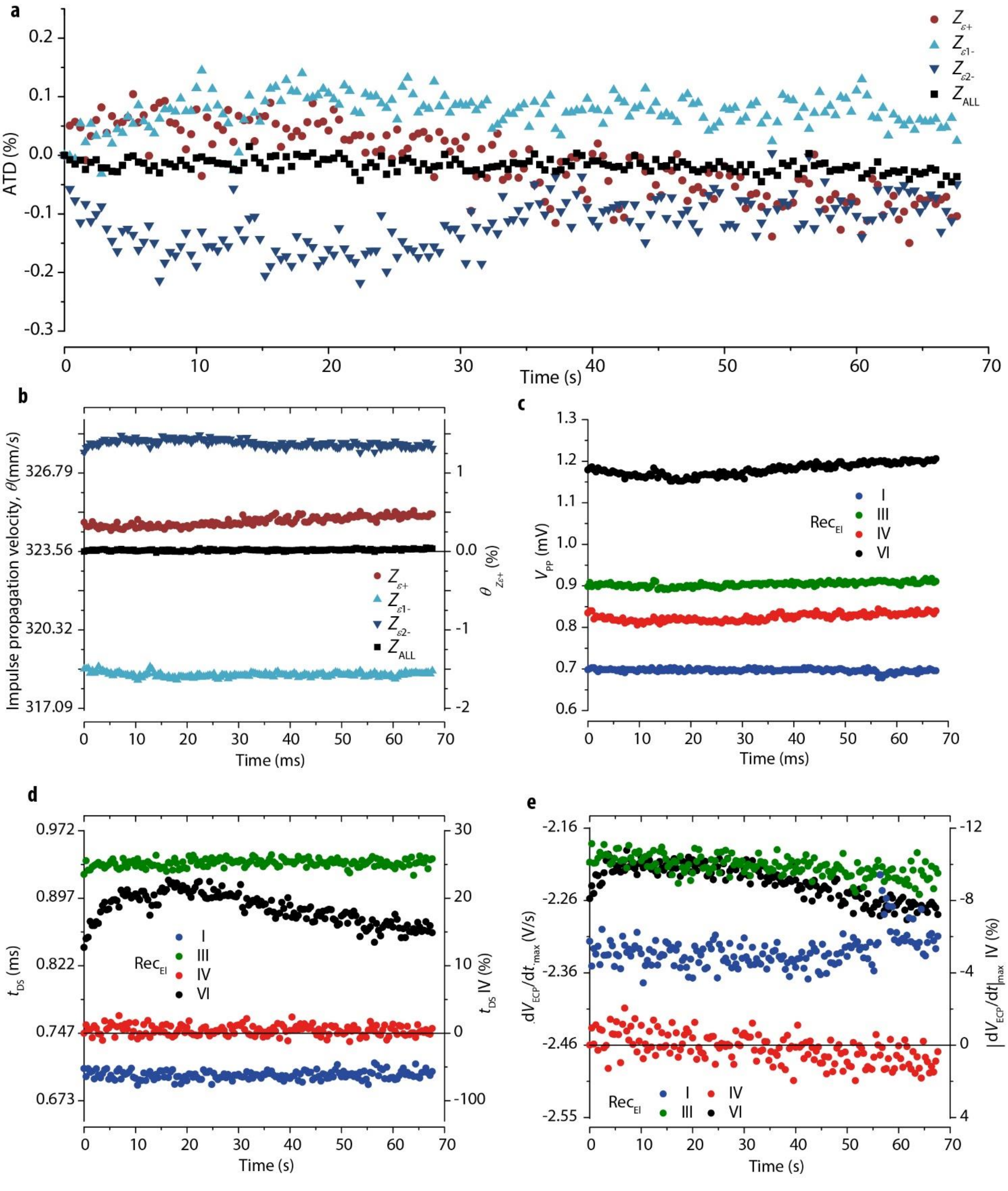

Supplementary Figure 8. AP_{EC} and conduction velocity stability under no strain conditions. **a)** Relative change in activation time difference measured overall and separately for the 3 strain zones. **b)** Impulse propagation velocity for the same regions as in a). Velocities differ slightly among the zones, showing a variation of $\pm$1.5% around the average overall velocity (black trace). **c)** V_{pp} varies significantly between Rec_{Els}, indicating possible differences in cell densities and electrical coupling to the Rec_{Els}. **d)** Downstroke times show little beat-to-beat scatter (~ 1%) and vary among Rec_{Els} which may be due to differences in electrode capacitance**. e)** Maximal downstroke velocities show a total spread of order of 10% with measurements by individual Rec_{Els} being stable to within 2%.

Rec_{EL} IV are included for the velocity, θ, (black data points in Supplementary Fig. 8b), the downstroke time, t_{DS}, (Supplementary Fig. 8d), and for the maximum downstroke velocity, $\left|\frac{dV_{\mathrm{ECP}}}{dt}\right|_{\max}$, (Supplementary Fig. 8e) The results show that V_{pp}, t_{DS} and maximal downstroke velocity of the AP_{ECs} are, with the exception of moderate drift present at Rec_{EI}VI, stable to within ±1% over the entire measurement period. Inter-electrode variability is largest for V_{pp}, which is the expected result because this parameter is sensitive to local conditions defining the coupling between the cell culture and the recording electrodes (such as local cell density, quality of the electrode, local contamination of the electrode by PDMS applied for insulation, etc.). On the other hand, ATDs and θ show minimal variations over time with scatter (peak-to-peak) being on the order of 0.1% and drift being less than 0.2% for all recordings. These small variations are corroborated by the finding that measurements over the entire width of the cell culture ($ATD_{I\text{-}VI}$ and $\theta_{I\text{-}VI}$) show the least variations. This indicates that the scatter observed is a result from changes in the actual impulse speed across the strand rather than noise in the specific timing measurements at the electrodes.

The observation that stable recordings of ATDs with detection errors <10 µs are feasible indicates that small changes in impulse conduction velocity during strain application can be tracked and quantified reliably. This is exemplified by the observation depicted in Fig. 4 of the main manuscript, where compressive strain towards the outer rim of the MaMEA results in measurable early arrival times at Rec_{EI}I as small as 40 µs.

Measurement stability in presence of strain is illustrated in **Supplementary Fig. 9** which depicts ATDs for the compressive and tensile zones of the MaMEA during application of strain protocols over a period > 50 min (data correspond to the data set shown in the main manuscript). Panel a) shows all data consisting of five sets of strains applied at increasingly faster strain rates with up to six different strain actuation amplitudes, V_ε, per given strain rate. The plot illustrates the following: (1) the overall drift of ATDs after 250 strain modulations (and > 50 min of experimental time) in respect to the initial measurement is less than 4%. Moreover, there is no systematic evidence that increasing strain amplitudes and strain rates have an adverse effect on the viability of the cell strand. To the contrary, after a slight and unexplained shift of ATDs following the initial slow strain protocol (200 ms ramp time), control ATDs measured at the beginning of each subsequent strain protocol remain stable throughout the remainder of the experiment. (2) The offset of ATDs present at the end of each series of strain modulations appears to largely relax during the ~ 1 min pause before the subsequent strain protocol is applied. To which extent this phenomenon is due

to relaxation processes within the PDMS membrane and whether short term and reversible alterations in cellular electrophysiology contribute to the build-up and relaxation of ATDs during the strain protocols remains to be shown. **Supplementary Fig. 9**b shows an excerpt of the data for t_{ramp} = 5 ms which illustrate a return to initial conditions within 0.25% over the six subsequent measurement protocols. These data suggest that, other than short-term memory effects, the CMC strand is not adversely affected by repeated mechanical perturbations.

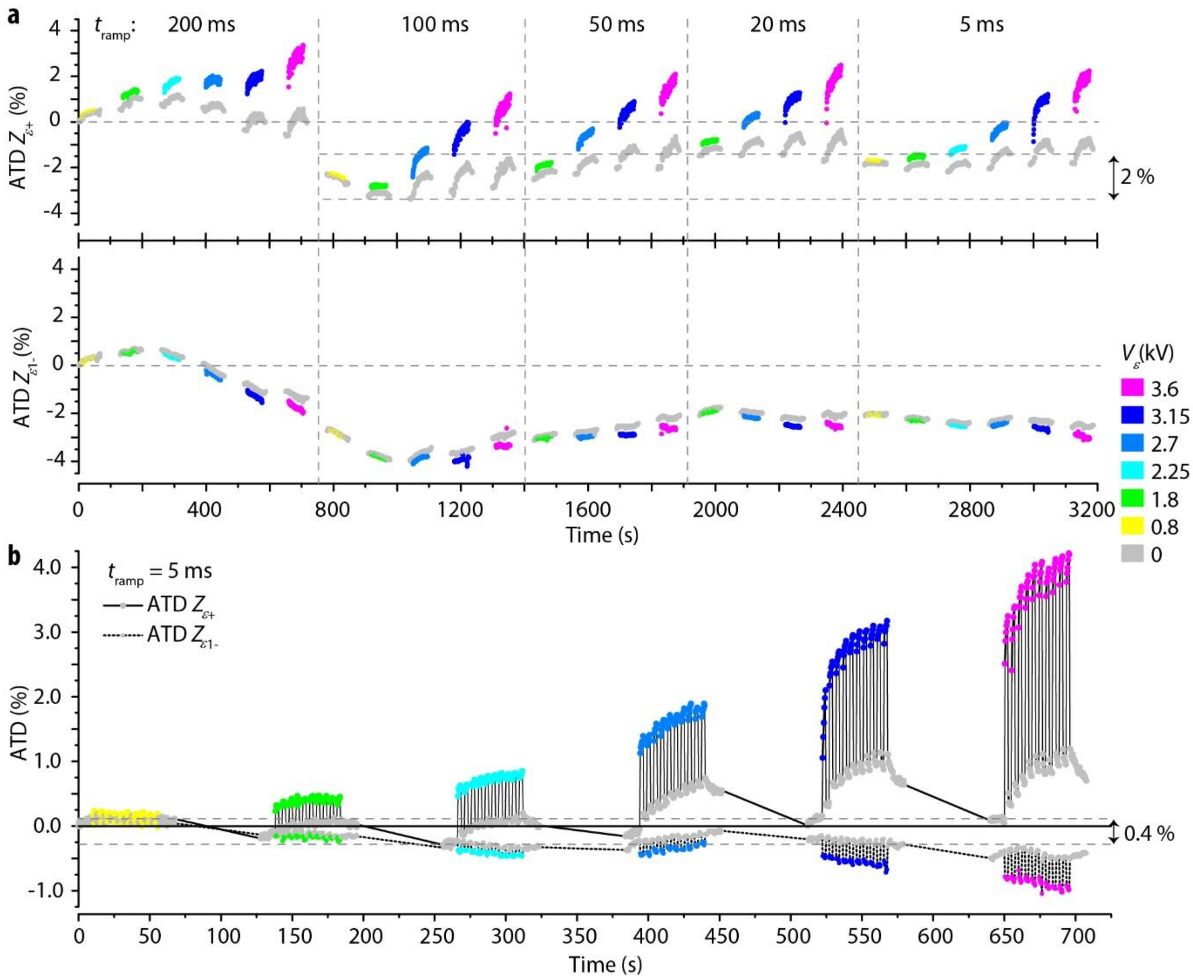

Supplementary Figure 9. ATD variations during application of strain protocols to a cardiomyocyte cell strand. **a)** ATD variations over the course of the entire series of strain modulations (different colors denote different strain amplitudes; vertical dashed lines separate protocols with increasing strain rates). The complete data set covers over 55 minutes of recordings with a total of 25’500 AP_{ECs} recorded by the six Rec_{Els}. **b)** Data obtained with a ramp time of 5 ms are replotted on an expanded time scale. For the $Z_{\varepsilon+}$ zone, baseline recording of ATDs obtained 10 s before the next strain modulation remain within 0.3 % of the very first measurement. For $Z_{\varepsilon-}$, ATDs baseline recordings decrease over time by ~ - 0.5%.

Supplementary Note 8: Detection of Action Potential Repolarization in Strands of Neonatal Rat Ventricular Cardiomyocytes and hi-PSC Derived Cardiomyocytes

To demonstrate the suitability of the MaMEA platform to record repolarization related extracellular signals in strand preparations of neonatal rat ventricular cardiomyocytes, we increased the coupling time constant of the AC-coupling circuit that connects the gold ion-implanted electrodes to the amplifiers. With the setting used for the experiments shown in this study (time constant, τ_c, of 8 ms), the fast initial signal caused by the action potential upstroke was followed by a stable baseline, i.e., the repolarization related signal was entirely suppressed by the high-pass filtering effect of the coupling circuitry (Supplementary Figure 10a). When increasing τ_c, to 1600 ms, the cut-on frequency of the circuitry was reduced accordingly and the

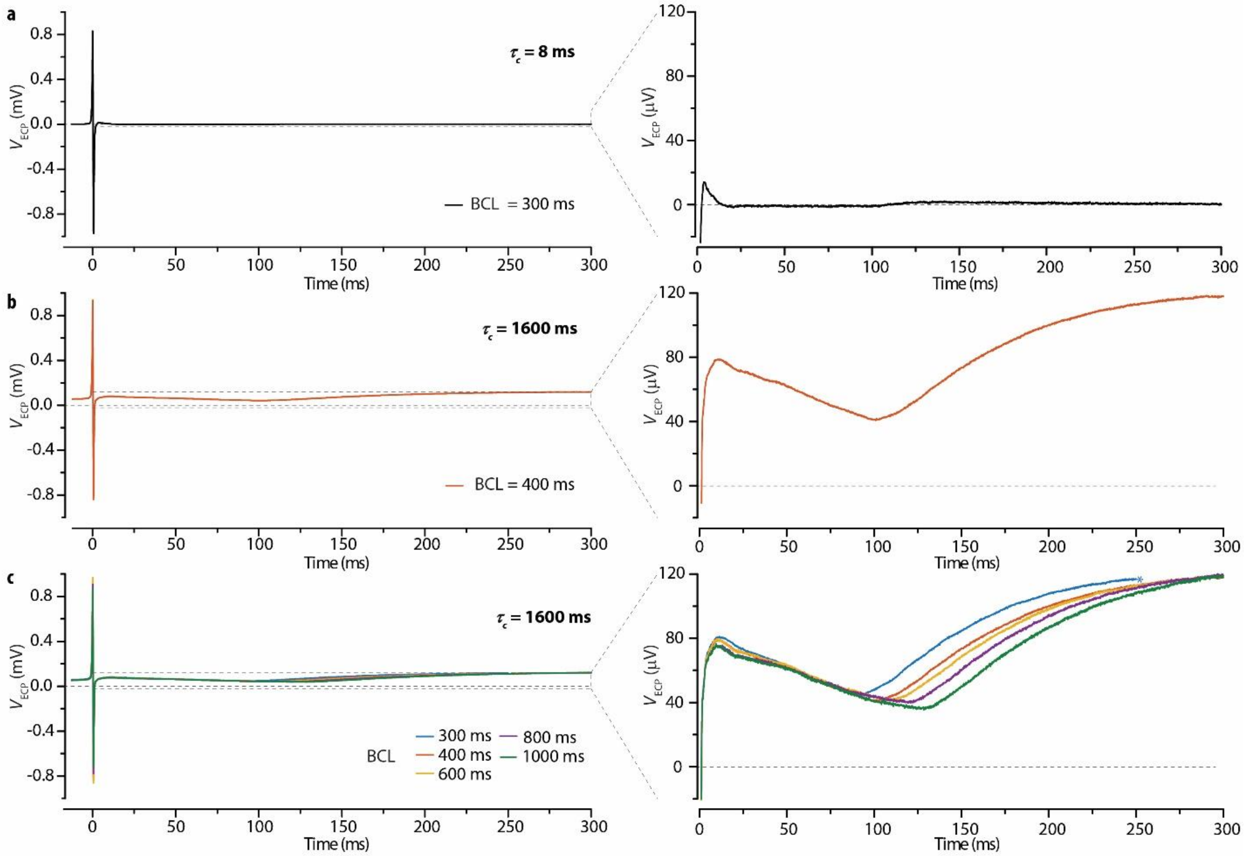

Supplementary Figure 10. Extracellular potentials of stimulated rat cardiomyocyte strand preparations recorded at different settings of the coupling time constants (τ_c) of the electronic circuit that connect the electrodes to the amplifiers. **a)** Extracellular signals recorded at a basic cycle length (BCL) of 300 ms with settings (τ_c = 8 ms) identical to those used in the experiments shown in the manuscript. While the initial action potential upstroke related signal is distinct (left panel), repolarization related signals are suppressed by the short τ_c even when magnifying the section of interest (right panel). **b)** Repolarization related extracellular signals are unveiled when τ_c is increased to 1600 ms, BCL = 400 ms. **c)** With increasing BCL, repolarization related field potentials are right-shifted which reflects *APD* restitution. (Blue star: truncation of signal to remove the artifact of the subsequent stimulation pulse. Traces: mean of 6 consecutive recordings).

repolarization signal was revealed (Supplementary Figure 10b). This signal followed without delay on the initial fast, upstroke-related signal which is in accordance to data presented by others and is to be expected given that the rat cardiomyocyte action potential has a rather triangular shape.[12] Functional proof that this signal was due to action potential repolarization was achieved by demonstrating presence of electrical restitution of action potential duration (Supplementary Figure 10c).

To furthermore demonstrate that the MaMEA system can be used with cell types different from rat cardiomyocytes, we generated strands consisting of hi-PSC derived cardiomyocytes. As shown in Supplementary Figure 11 and similar to rat cardiomyocytes, strands constructed from these stem-cell

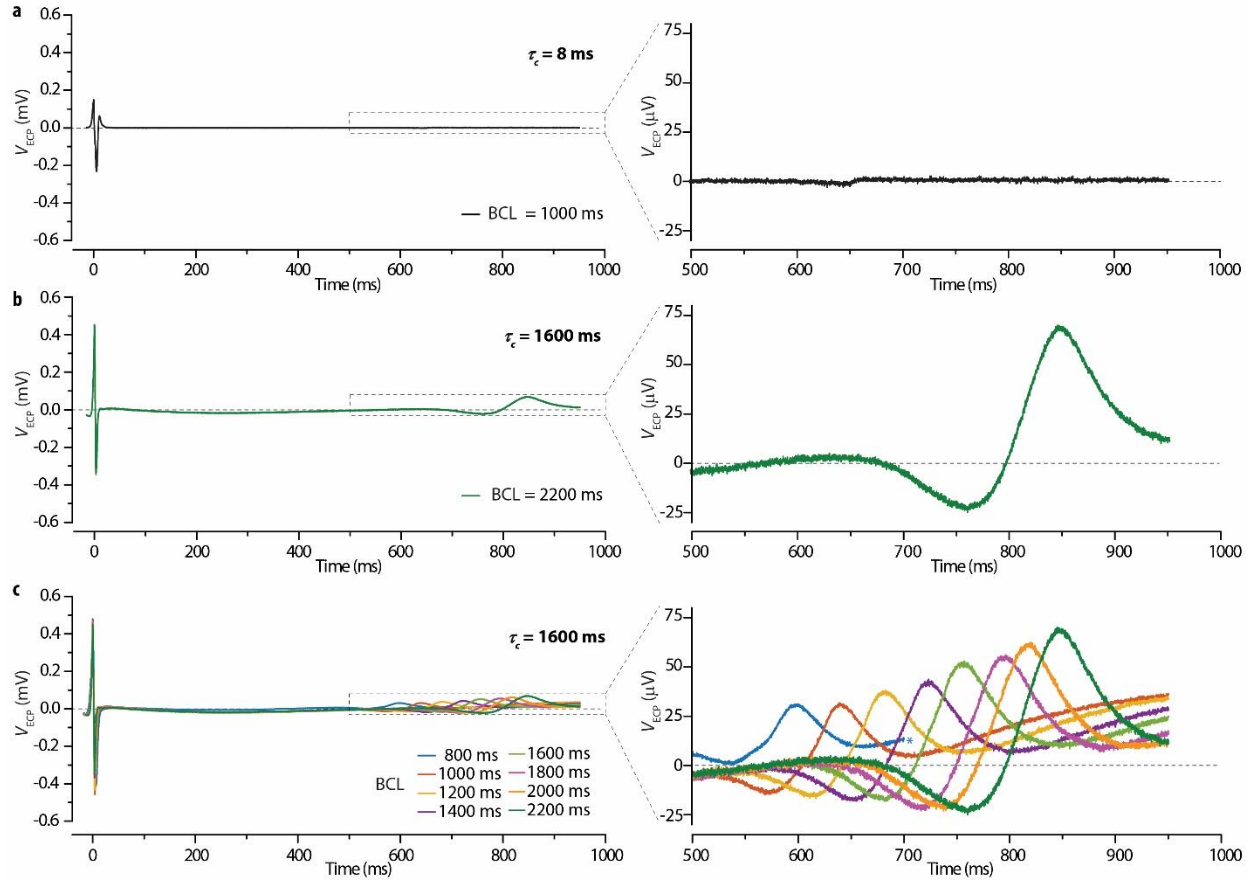

Supplementary Figure 11. Extracellular potentials of stimulated strands of hi-PSC derived cardiomyocytes. **a)** Extracellular signals recorded at a basic cycle length (BCL) of 300 ms with τ_c = 8 ms. While the initial action potential upstroke related signal is distinct (left panel), repolarization related signals are suppressed by the short τ_c even when magnifying the section of interest (right panel). **b)** Repolarization related extracellular signals are unveiled when τ_c is increased 1600 ms. **c)** With increasing BCL, repolarization related field potentials are right-shifted which reflects *APD* restitution. (Blue star: truncation of signal to remove the artifact of the subsequent stimulation pulse. Traces: mean of 6 consecutive recordings).

derived cardiomyocytes produced fast signals related to the action potential upstroke. When increasing τ_c, from 8 ms to 1600 ms, the initial fast signal was followed by a second biphasic signal representing action

potential repolarization which was again functionally validated by showing presence of action potential duration restitution. In contrast to the rat cardiomyocyte repolarization signal following immediately after the first fast signal, the repolarization related signal of stem-cell derived cardiomyocytes occurred with a distinct delay that reflects the extended plateau phase of these cells.[13,14]

Supplementary Note 9: Deriving $\boldsymbol{\theta}$ from Activation Time Differences

The data presented in Fig 5b show that activation time differences (ATDs) scale linearly with applied mechanical strain. *ie*:

$$\Delta\mathrm{ATD}(\varepsilon) = \frac{\mathrm{ATD}(\varepsilon) - \mathrm{ATD}_0}{\mathrm{ATD}_0} = \alpha\varepsilon, \qquad \text{(S5)}$$

with α being a unitless parameter that was experimentally determined to equal 0.31 for the preparation shown in Fig 5b. From this, one can extract the scaling behavior of θ given that:

$$\theta = \frac{\Delta x}{\Delta t}, \qquad \text{(S6)}$$

where Δx is the distance between two measurement points and Δt the difference of the respective activation times. From supplementary equation (S5), the scaling of ATD with regards to strain ε is

$$\Delta t = \mathrm{ATD}(\varepsilon) = \alpha\varepsilon\mathrm{ATD}_0 + \mathrm{ATD}_0 = (\alpha\varepsilon + 1)\Delta t_0. \qquad \text{(S7)}$$

Given that distance scales with strain as $\Delta x = (\varepsilon + 1)\Delta x_0$, then θ scales with strain as

$$\theta = \frac{\Delta x}{\Delta t} = \frac{(\varepsilon+1)\Delta x_0}{(\alpha\varepsilon+1)\Delta t_0} = \frac{(\varepsilon+1)}{(\alpha\varepsilon+1)}\theta_0,$$

$$\frac{\theta(\varepsilon)}{\theta_0} \approx \left(1 + \left(\frac{1}{\alpha} - 1\right)(\alpha\varepsilon - \alpha^2\varepsilon^2 + O(\varepsilon^3))\right) \qquad \text{(S8)}$$

Regarding the data set presented in the main manuscript (ε<0.12 and $\alpha \approx 0.3$), including up to the quadratic term of the series expansion is sufficient to describe the data, with higher order terms being well within the uncertainty of the measurements.

Supplementary Note 10: Cable Equation Applied to a Linearly Strained Cell Strand

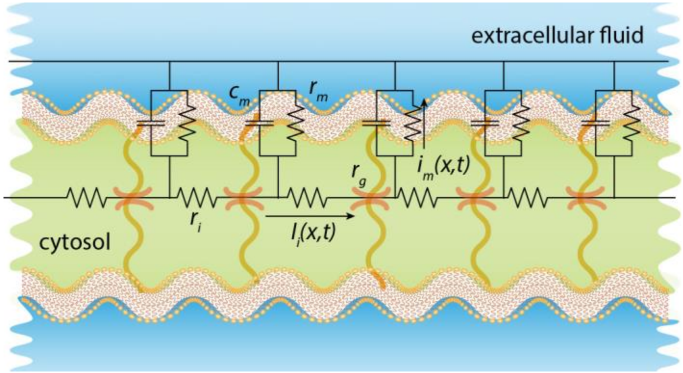

Supplementary Figure 12. Passive model of a strand of cardiomyocytes.

Here we consider how the Cable Theory predicts the scaling of impulse propagation velocity with regards to strain. Supplementary Fig. 13 depicts a simplified model of a chain of cardiomyocytes where only passive elements (*i.e.* cytosolic resistance per unit length, r_i [Ω/m], gap junction resistance per unit length, r_g, [Ω/m] cell membrane resistance, $\tilde{r}_m$ [Ω m], and cell membrane capacitance per unit length, c_m [F/m]) are considered. Ion channels, pumps and exchangers are not included, and extracellular longitudinal resistance is assumed to be negligible. For this simplified case, the cable equation can be expressed as

$$\frac{\tilde{r}_m}{(r_i+r_g)}\frac{\partial^2 V(x)}{\partial x^2} - \tilde{r}_m c_m \frac{\partial V(x,t)}{\partial t} - V(x,t) = 0,$$

$$\lambda^2 \frac{\partial^2 V(x)}{\partial x^2} - \tau \frac{\partial V(x,t)}{\partial t} - V(x,t) = 0, \qquad \text{(S9)}$$

Supp. eqn. (S9) defines the length constant $\lambda = \sqrt{\frac{\tilde{r}_m}{r_i+r_g}}$ [m] and the time constant $\tau = \tilde{r}_m c_m$ [s].

Because the length constant λ scales with the velocity of the impulse propagation and τ relates to the time needed for eliciting an action potential, conduction velocity can be defined as $\theta \approx 2\frac{\lambda}{\tau}$ (for $x \gg \lambda$, following Dayand an Abbott[15] where the solution for the infinite cable is derived).

By considering how the passive elements respond to stretch, one can derive scaling laws for λ and τ, and hence for θ. The predicted strain dependences are summarized in Supplementary Table 1:

Relaxed parameter	Stretched parameter $\lambda_\varepsilon\!=\!\varepsilon\!+\!1$	Notes/Explanations
r_i	$\lambda_\varepsilon r_i$	Scales as 1/ the cross sectional area of the cell strand (resistances in series)
r_g	$\frac{r_g}{\lambda_\varepsilon}$	Scales with the density of gap junction channels (resistances in series)
$\tilde{r}_m$	$\lambda_\varepsilon \tilde{r}_m$	Scales with the length (resistances in parallel)
c_m	$\frac{c_m}{\lambda_\varepsilon}$	Scales as capacitance in parallel
$\lambda = \sqrt{\frac{\tilde{r}_m}{r_i+r_g}} = \sqrt{\frac{\tilde{r}_m}{r_l(x+(1-x))}}$	$\frac{\sqrt{\lambda_\varepsilon}}{\sqrt{\lambda_\varepsilon x+\frac{(1-x)}{\lambda_\varepsilon}}}\lambda$	$x=\frac{r_i}{r_l}$ is the weight of cytosolic resistance per unit length compared to the total longitudinal resistance per unit length $r_l = r_i + r_g$
$\tau = \tilde{r}_m c_m$	τ	Stretch independent! (in agreement with the observation of an invariant t_{DS}).
θ	$\frac{\sqrt{\lambda_\varepsilon}}{\sqrt{\lambda_\varepsilon x+\frac{(1-x)}{\lambda_\varepsilon}}}\theta$	

Supplementary Table 1 Scaling laws of a stretched cell strand. Stretch $\lambda_\varepsilon = 1 + \varepsilon$, is used as the scaling parameter. A folded cell membrane (constant surface area) is assumed.

Given these scaling laws, τ becomes independent of strain which is supported by the finding that maximal downstroke times and velocities were not or only barely affected by strain. Hence, the velocity scales with the length constant λ. By matching the theory to the data, one can determine the relative contribution of the cytosolic and the gap junctional resistance by considering the only remaining free parameter, *x*.

Comparing theory with experimental results:

The derived cable theory and the empirically found results can be compared after a series expansion (around ε = 0) of supp. eqn. (S8) and the result is summarized in Supplementary Table 1.

$$\frac{\theta(\varepsilon)}{\theta_0}_{experiment} = \frac{(\varepsilon+1)}{(\alpha\varepsilon+1)} \approx \left(1 + \left(\frac{1}{\alpha} - 1\right)\left(\alpha\varepsilon - \alpha^2\varepsilon^2 + O(\varepsilon^3)\right)\right) = 1 + 0.70\,\varepsilon - 0.21\varepsilon^2 - O(10^{-5}). \quad \text{(S10)}$$

$$\frac{\theta(\varepsilon)}{\theta_0}_{Calbe\ Theory} = \frac{\sqrt{\lambda_\varepsilon}}{\sqrt{\lambda_\varepsilon x+\frac{(1-x)}{\lambda_\varepsilon}}} \approx 1 + (1-x)\varepsilon + \frac{3}{2}x(x-1)\varepsilon^2 + O(\varepsilon^3). \quad \text{(S11)}$$

where *x* (the ratio of cytosol and gap junction) is a free parameter and is chosen to fit the experimental results. Hence, including the first two terms and assuming a strain of 10% one can find *x* by supplementary equating (S11) with (S10):

$$(1-x)\varepsilon + \frac{3}{2}x(x-1)\varepsilon^2 = \left(\frac{1}{\alpha} - 1\right)(\alpha\varepsilon - \alpha^2\varepsilon^2)$$

$$x = \frac{2 + 3\varepsilon - \sqrt{4 + 12(1-2\alpha)\varepsilon + (9 - 24\alpha(1-\alpha))\varepsilon^2}}{6\varepsilon} \qquad \text{(S12)}$$

$$= 0.29\ (\varepsilon = 0.1, \alpha = 0.304).$$

This analysis suggests that, for the preparation described in the main manuscript, the increase of ATDs during strain can be explained solely by the deformation induced increase in axial resistance if the cytosolic and gap junctional resistance contribute 29% and 71%, respectively, to overall axial resistance. These numbers are between generally assumed relative contributions (50/50%)[16] and recently published values using cultured cardiomyocyte strands (25/75%)[7]. While this does not a priori rule out additional contributions from stretch activated channels, changes in cell capacitance and effects related to supernormal conduction, these contributions seem to be minor.

Supplementary Note 11: Effects of Strain on Impulse Conduction in Cardiomyocyte Cell Strands - Additional Experiments

To validate the results presented in the main manuscript, identical experiments were performed with 5 additional preparations cultured in 4 different devices with the results being shown in Supplementary Fig. 13b-f (Source data are provided[17]). Supplementary Fig. 13a reproducing the findings presented in the main manuscript for comparison purposes. For each preparation, ATD vs. strain rate, ATD vs. strain, and θ vs. strain is plotted in three adjacent panels. The additional experiments permit to draw the following conclusions: (1) for any given strain amplitude, ATDs are largely independent of strain rates up to highly supraphysiological levels (~ 29 /s) thereby corroborating the main findings presented in the manuscript. (2) ATD dependence on strain amplitude, α, was determined to be 0.31 (Supplementary Fig. 13a), 0.29 (Supplementary Fig. 13b), 0.43 (Supplementary Fig. 13c), 0.38 (Supplementary Fig. 13d), 0.32 (Supplementary Fig. 13e), and 0.37 (Supplementary Fig. 13f) thus covering a relatively narrow range.

Overall, the measurements demonstrate that, while being dependent on strain amplitude in a cable-theory dependent manner, impulse conduction is not affected by increasing the strain rate to highly supraphysiological levels. Future experiments will have to show whether this insensitivity is lost at even higher strain rates (it is feasible to reach up to 1000 /s with the system presented using appropriate DEA drivers[11]) or if strain rate sensitivity is altered in presence of electronically coupled myofibroblasts.[18]

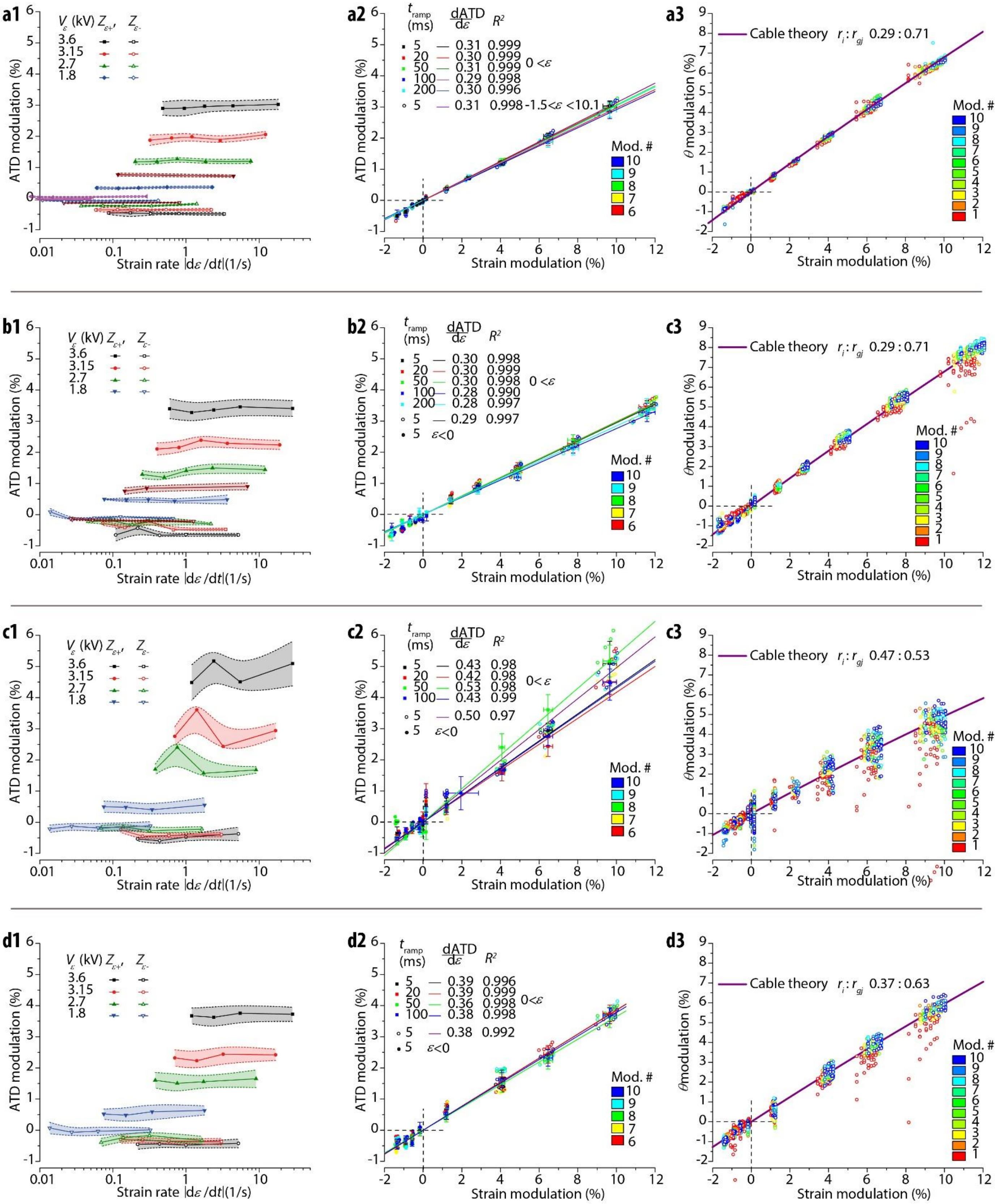
a1
a2
a3
b1
b2
c3
c1
c2
c3
d1
d2
d3
ATD modulation (%)
θ modulation (%)
Strain rate |dε/dt|(1/s)
Strain modulation (%)
Vε (kV) Zε+, Zε-
3.6
3.15
2.7
1.8
t ramp (ms)
dATD/dε
R²
0 < ε
-1.5< ε <10.1
ε<0
Cable theory ri : rgj 0.29 : 0.71
Cable theory ri : rgj 0.47 : 0.53
Cable theory ri : rgj 0.37 : 0.63
Mod. #

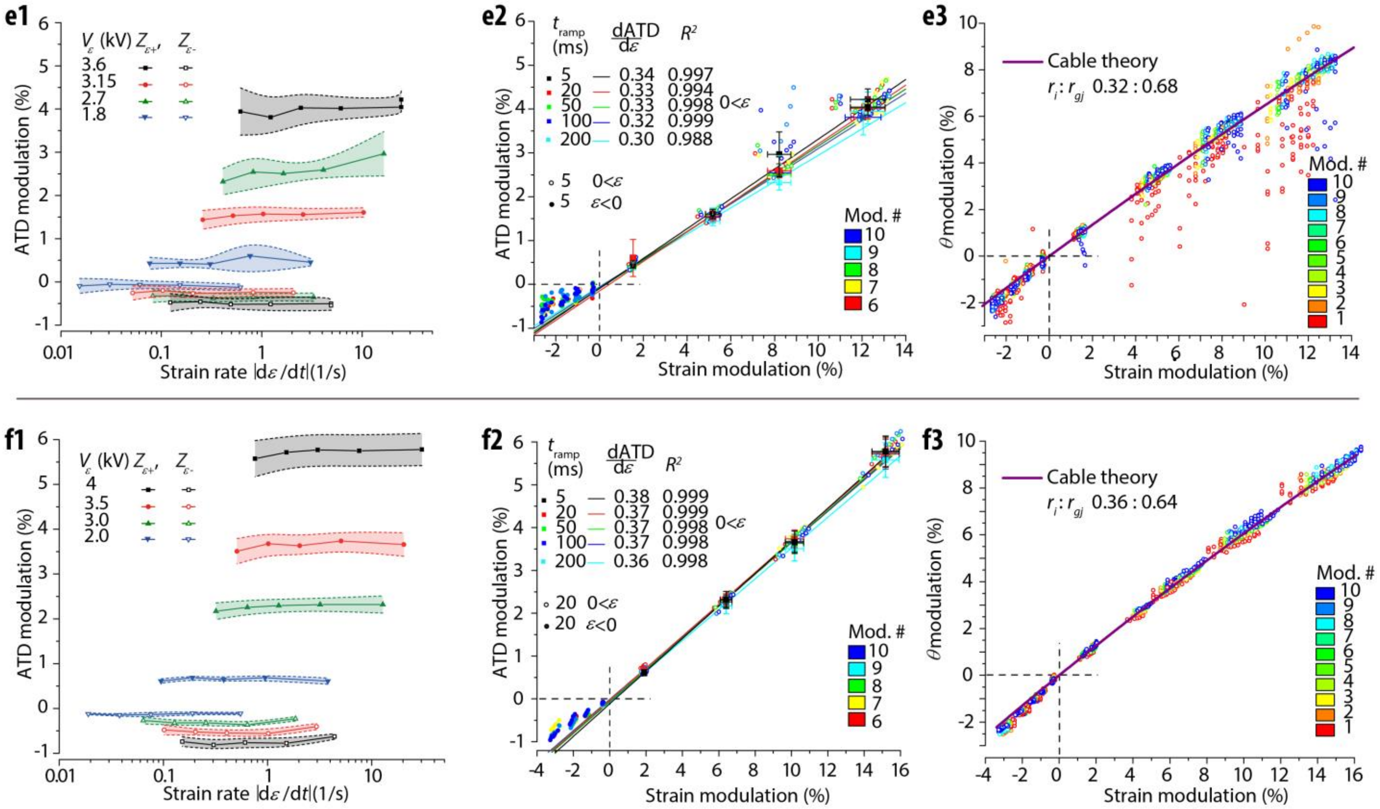

Supplementary Figure 13. Replication of the experiments shown in the manuscript with 5 independent additional preparations subjected to identical strain amplitude - strain rate protocols. Data are presented according to Figure 5 of the manuscript which is reproduced for comparison purposes (panels a1-a3). The left panels depict changes in activation time difference (ATD) as a function of different strain rates and strain amplitudes corresponding to specific DEA drive voltages (color coded). The middle panels show the dependence of ATDs on strain modulation for different strain rates (color coded) whereas the right panels show the same data after transformation of the ATD data ('preparation coordinates') into conduction velocity data ('observer coordinates', i.e., distance) with values being fitted by cable equations that take into account myoplasmic (r_i) and gap junctional (r_{gj}) resistivity. Source data are provided.

Supplementary Note 12: Control Experiments with Mechanically Immobilized PDMS Membranes

To test whether exposure to high voltage fields affects the electrical function of cultured cardiomyocytes in the absence of mechanical strain, the underside of the PDMS membrane of MaMEAs was mechanically stabilized with Mylar before subjecting the cultures to standard high voltage protocols. This type of immobilization has previously been described.[5] Optical control measurements confirmed that there was no strain development in presence of Mylar. As shown in Supplementary Fig. 14, applying high voltage pulses

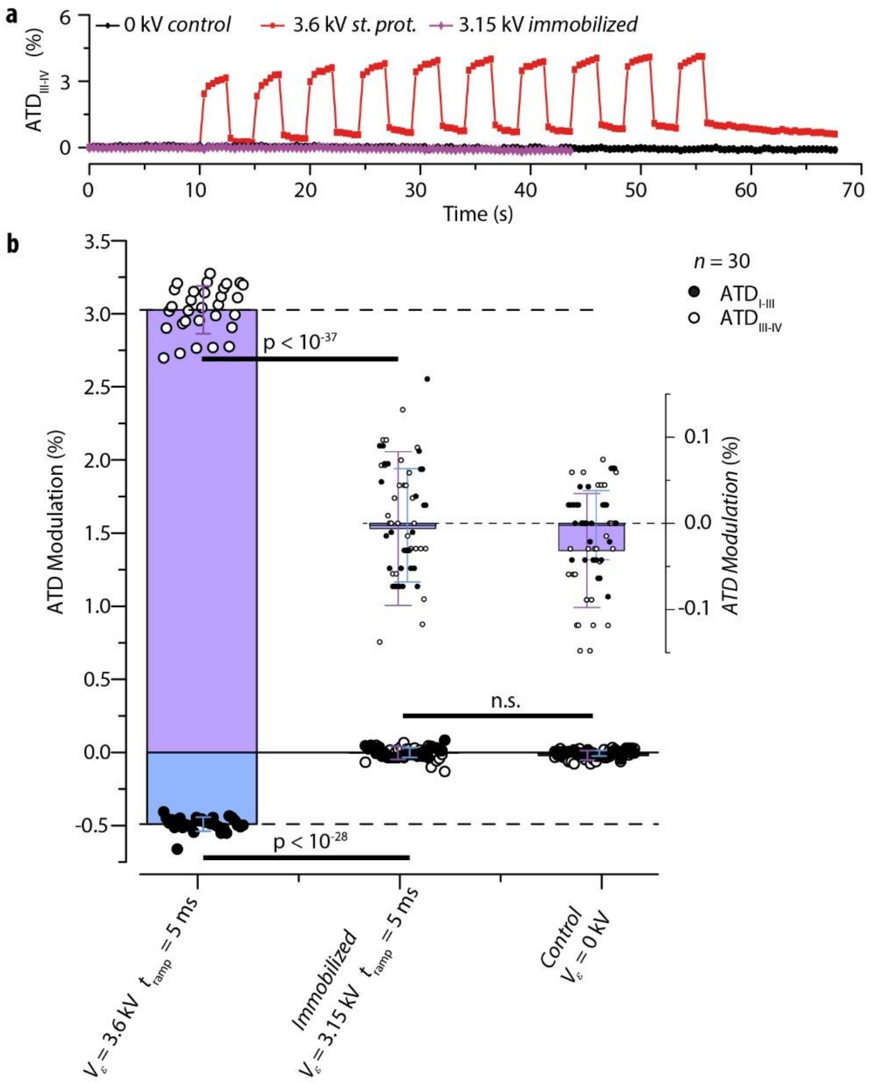

Supplementary Figure 14. Effect of high voltage electrical fields on impulse conduction. **a)** Comparison of baseline measurement without DEA actuation (black, V_ε = 0 kV), measurements using the standard strain protocol (red, V_ε = 3.6 kV, t_{ramp} = 5 ms) and measurements with mechanically immobilized DEAs being electrically activated (purple, V_ε = 3.15 kV, t_{ramp} = 5 ms, $\varepsilon \equiv 0$). **b)** Summary of results with inclusion of measurements in the negative strain zone and with ATD modulation being corrected for drift. Standard deviation indicated by error bars (*n*=30 each).

to mechanically immobilized DEAs had no measurable effect on impulse conduction as demonstrated by the complete overlap of these data (purple trace: this trace contains 5 instead of the standard 10 strain modulations, which was deemed sufficient as no change in ATDs could be observed) to those obtained in absence of DEA activation (black trace). Upon release of the mechanical immobilization, preparations showed normal modulation of ATDs with regards to modulated strain (red trace). The summary data shown in Supplementary Fig. 14b confirm that ATDs measured in the absence of DEA actuation and ATDs measured in presence of electrically driven but mechanically immobilized membranes were similar. This

finding is relevant if DEAs are to be used as motors for mechanical modulation because it eliminates the concern that the observations are affected by the high electrical fields produced by DEAs.

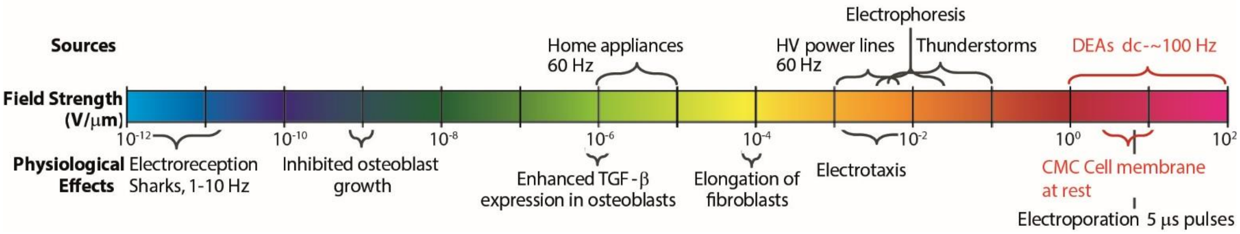

Supplementary Figure 15. Physiological effects of electrical fields. Top: Typical non-biological sources of electrical fields, bottom: physiological effects observed.

Supplementary Note 13: Effects of High Electrical Field Strengths on Cardiomyocytes

The field strength in a DEA can reach 10^2 V/µm which may cause concerns regarding its effect on cardiomyocytes close by. Although most of the field is shielded by the ground electrodes, one would expect the fringe fields in close proximity to the edge of the DEA to be still significant (~1 V/µm). The literature provides little information on the effect of such fields on excitable cells as most experiments refer to fields within the culture medium during current flow. In our case, the field is only capacitively coupled to the cells with exchange of charge carriers occurring exclusively during the up and down ramps of V_ε. Furthermore, the presence of the conductive cell culture medium is expected to shield the time varying fields from reaching the cardiomyocytes.

Supplementary Fig. 15 compares typical environmental field strength to those caused by the DEA. Of note, cell membranes of cardiomyocytes at rest withstand very high fields in the order of 10 V/µm. As is evident in the figure, this does not exclude that smaller external fields can affect cell behavior as well.

To determine whether capacitively coupled electrical fields adversely affect cell viability, cardiomyocytes were grown on PDMS (100 µm thick) with gold electrodes having the same geometry as the DEAs being patterned beneath the PDMS, which permitted a direct capacitive coupling of the cells to the electrodes, hence leading to their exposure to full fields (not only fringe fields as present in the DEAs).

From 9 cultures, 3 served as controls while 6 were exposed to modulated electrical fields (1 Hz, 4 kV) for 6 hours. Cell viability was assessed with a Biotium Kit (PI-#30066) that can differentiate between apoptotic and necrotic cells. As illustrated by the phase contrast and fluorescence images in in Supplementary Fig. 16, exposure to modulated electrical fields affected neither the gross morphology of the cardiomyocyte monolayers nor did it cause obvious changes in the density of apoptotic and necrotic cells. This is in line

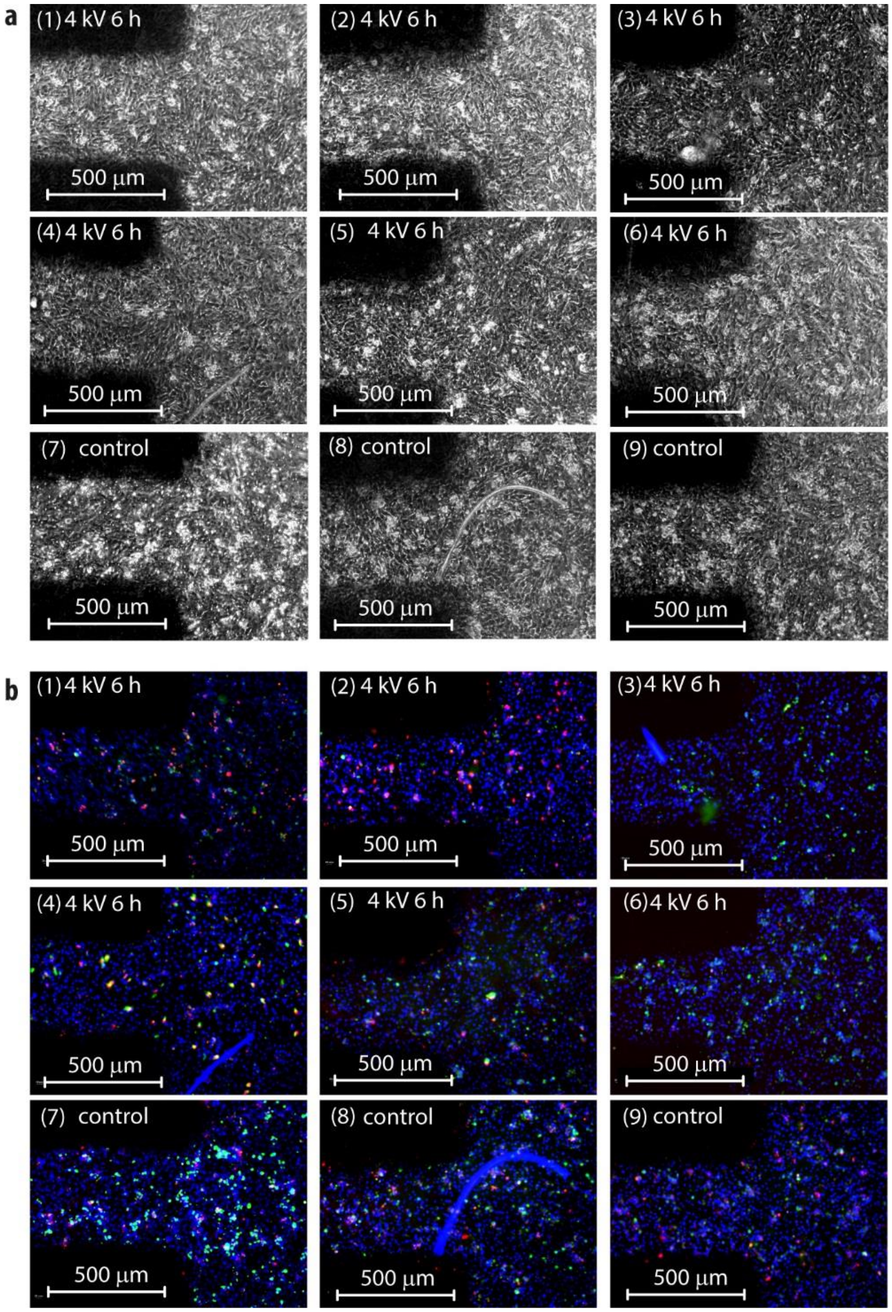

Supplementary Figure 16. Effects of modulated high voltage fields on cardiomyocyte viability. a) Phase contrast images of control and experimental preparations (black: gold electrodes. b) Fluorescence images of the same preparations depicting nuclei (blue, DAPI), apoptotic cells (green) and necrotic cells (red).

with the findings presented in the main manuscript that demonstrate that function in respect to θ and AP_{EC} characteristics was not affected by prolonged exposure to (admittedly smaller) electrical fields.

A further question in the context of fast changing electrical fields concerns their ability to trigger electrical activity. Such triggering was occasionally observed in the test cultures shown in Supplementary Fig. 16. By

contrast, field triggering was not observed in the strand preparations which is likely due to the fact that fields were smaller (fringe fields) and the rise time of the field was substantially slower (5 ms vs. 10 µs).

Supplementary References

1. Pelrine, R., Kornbluh, R., Pei, Q. & Joseph, J. High-speed electrically actuated elastomers with strain greater than 100%. *Science (80-)* 287, 836–839 (2000).
2. Rosset, S. & Shea, H. R. Flexible and stretchable electrodes for dielectric elastomer actuators. *Appl Phys A* 110, 281–307 (2012).
3. Akbari, S. & Shea, H. R. An array of 100 µm × 100 µm dielectric elastomer actuators with 80% strain for tissue engineering applications. *Sensors Actuators, A Phys* 186, 236–241 (2012).
4. Akbari, S., Rosset, S. & Shea, H. R. Improved electromechanical behavior in castable dielectric elastomer actuators. *Appl Phys Lett* 102, 071906 (2013).
5. Poulin, A., Saygili Demir, C., Rosset, S., Petrova, T. V. & Shea, H. Dielectric elastomer actuator for mechanical loading of 2D cell cultures. *Lab Chip* 16, 3788–3794 (2016).
6. Rosset, S., Araromi, O. A., Schlatter, S. & Shea, H. R. Fabrication Process of Silicone-based Dielectric Elastomer Actuators. *J Vis Exp* e53423 (2016). doi:10.3791/53423
7. Buccarello, A., Azzarito, M., Michoud, F., Lacour, S. P. & Kucera, J. P. Uniaxial strain of cultured mouse and rat cardiomyocyte strands slows conduction more when its axis is parallel to impulse propagation than when it is perpendicular. *Acta Physiol* 1–19 (2018). at <http://doi.wiley.com/10.1111/apha.13026>
8. Poulin, A., Martin-Olmos, C., Rosset, S., Liley, M. & Shea, H. DEA-based deformable cell culture system. (2014). at <http://infoscience.epfl.ch/record/214516>
9. Rosset, S., Niklaus, M., Dubois, P. & Shea, H. R. Metal ion implantation for the fabrication of stretchable electrodes on elastomers. *Adv Funct Mater* 19, 470–478 (2009).
10. Poulin, A. Miniaturized Dielectric Elastomer Actuator for Mechanical Stimulation of Monolayer Cell Cultures. 126 (2016).
11. Poulin, A. *et al.* An ultra-fast mechanically active cell culture substrate. *Sci Rep* 8, (2018).

12. Uchida, T., Kitora, R. & Gohara, K. Temperature dependence of synchronized beating of cultured neonatal rat heart-cell networks with increasing age measured by multi-electrode arrays. *Res Artic Trends Med* 18, 1–10 (2018).

13. Asakura, K. *et al.* Improvement of acquisition and analysis methods in multi-electrode array experiments with iPS cell-derived cardiomyocytes. *J Pharmacol Toxicol Methods* 75, 17–26 (2015).

14. Tertoolen, L. G. J., Braam, S. R., van Meer, B. J., Passier, R. & Mummery, C. L. Interpretation of field potentials measured on a multi electrode array in pharmacological toxicity screening on primary and human pluripotent stem cell-derived cardiomyocytes. *Biochem Biophys Res Commun* 497, 1135–1141 (2018).

15. Dayan, P. & Abbott, L. F. *Theoretical neuroscience*. 806, (Cambridge, MA: MIT Press, 2001).

16. Fast, V. G. & Kléber, A. G. Microscopic conduction in cultured strands of neonatal rat heart cells measured with voltage-sensitive dyes. *Circ Res* 73, 914–25 (1993).

17. Imboden, M. *et al.* Source data underlying Fig. 5 and Supplementary Fig. 13. (2018). doi:10.5281/zenodo.2541654

18. Grand, T., Salvarani, N., Jousset, F. & Rohr, S. Aggravation of cardiac myofibroblast arrhythmogeneicity by mechanical stress. *Cardiovasc Res* 104, 489–500 (2014).
