# Supplementary material for: High-speed mechano-active multielectrode array for investigating rapid stretch effects on cardiac tissue
Source: Nat Commun. 2019 Feb 19;10:834. doi: 10.1038/s41467-019-08757-2 (PMC6381132; doi:10.1038/s41467-019-08757-2)
Supplement: Supplementary file 2 — Description of Additional Supplementary Files [file 41467_2019_8757_MOESM2_ESM.docx]

**Description of Additional Supplementary Files**

File Name: Supplementary Movie 1

Description: The supplementary movie illustrates the key concepts of the work. The experimental setup, based on the Mechanically Active Multi Electrode Array is outlined, followed by a conceptualization of the experiments performed. Then the underlying measurements and conclusions, illustrating the insensitivity of impulse conduction with regards to the applied strain rate, is presented.
