# Supplementary material for: High-speed mechano-active multielectrode array for investigating rapid stretch effects on cardiac tissue
Source: Nat Commun. 2019 Feb 19;10:834. doi: 10.1038/s41467-019-08757-2 (PMC6381132; doi:10.1038/s41467-019-08757-2)
Supplement: Supplementary file 4 — Reporting Summary [file 41467_2019_8757_MOESM4_ESM.pdf]

nature research

Corresponding author(s): Matthias Imboden

Last updated by author(s): Jan 12, 2019

Reporting Summary

Nature Research wishes to improve the reproducibility of the work that we publish. This form provides structure for consistency and transparency in reporting. For further information on Nature Research policies, see Authors & Referees and the Editorial Policy Checklist.

Statistics

For all statistical analyses, confirm that the following items are present in the figure legend, table legend, main text, or Methods section.

n/a	Confirmed	
☐	☒	The exact sample size (*n*) for each experimental group/condition, given as a discrete number and unit of measurement
☐	☒	A statement on whether measurements were taken from distinct samples or whether the same sample was measured repeatedly
☒	☐	The statistical test(s) used AND whether they are one- or two-sided<br>*Only common tests should be described solely by name; describe more complex techniques in the Methods section.*
☒	☐	A description of all covariates tested
☒	☐	A description of any assumptions or corrections, such as tests of normality and adjustment for multiple comparisons
☒	☐	A full description of the statistical parameters including central tendency (e.g. means) or other basic estimates (e.g. regression coefficient) AND variation (e.g. standard deviation) or associated estimates of uncertainty (e.g. confidence intervals)
☒	☐	For null hypothesis testing, the test statistic (e.g. *F*, *t*, *r*) with confidence intervals, effect sizes, degrees of freedom and *P* value noted<br>*Give P values as exact values whenever suitable.*
☒	☐	For Bayesian analysis, information on the choice of priors and Markov chain Monte Carlo settings
☒	☐	For hierarchical and complex designs, identification of the appropriate level for tests and full reporting of outcomes
☒	☐	Estimates of effect sizes (e.g. Cohen's *d*, Pearson's *r*), indicating how they were calculated

Our web collection on statistics for biologists contains articles on many of the points above.

Software and code

Policy information about availability of computer code

Data collection	Data was collected with custom written software that controlled the custom developed strain device described in our study. The essential features of the hardware are described in detail in the supplement.
Data analysis	Data were analyzed using standard the standard software packages Matlab, Origin, and NI Visual Assistant. The analysis did not requore specific custom algorithms

For manuscripts utilizing custom algorithms or software that are central to the research but not yet described in published literature, software must be made available to editors/reviewers. We strongly encourage code deposition in a community repository (e.g. GitHub). See the Nature Research guidelines for submitting code & software for further information.

Data

Policy information about availability of data

All manuscripts must include a data availability statement. This statement should provide the following information, where applicable:

- Accession codes, unique identifiers, or web links for publicly available datasets
- A list of figures that have associated raw data
- A description of any restrictions on data availability

Data Availability: Source data underlying Fig. 5 (manuscript) and Supplementary Figure 13 are provided as a Source Data file (DOI:10.5281/zenodo.2541654. All other relevant data are available from the authors.)

Field-specific reporting

Please select the one below that is the best fit for your research. If you are not sure, read the appropriate sections before making your selection.

☒ Life sciences ☐ Behavioural & social sciences ☐ Ecological, evolutionary & environmental sciences

For a reference copy of the document with all sections, see nature.com/documents/nr-reporting-summary-flat.pdf

Life sciences study design

All studies must disclose on these points even when the disclosure is negative.

Sample size	Verification of the new experimental strain system described in the study was performed with 6 independent samples.
Data exclusions	To be included in the experimental analysis, the following criteria had to be met by the preparations: (i) ≥ 5 recording electrodes were operational (ii) the DEA actuator was able to generate ≥ 10% strain without breakdown, and (iii) cell strands displayed a uniform structure and displayed continuous conduction under control conditions. These conditions are listed in the Methods section of the manuscript.
Replication	The results obtained and described in the manuscript were replicated 5 times and are shown in the Supplement.
Randomization	The biological data presented are used to illustrate the capabilities of the new ultra-fast strain device and, hence, no randomization was necessary.
Blinding	As for randomization: the biological data presented are used to illustrate the capabilities of the new ultra-fast strain device and, hence, no blinding was necessary.

Reporting for specific materials, systems and methods

We require information from authors about some types of materials, experimental systems and methods used in many studies. Here, indicate whether each material, system or method listed is relevant to your study. If you are not sure if a list item applies to your research, read the appropriate section before selecting a response.

Materials & experimental systems

n/a	Involved in the study	
☒	☐	Antibodies
☒	☐	Eukaryotic cell lines
☒	☐	Palaeontology
☐	☒	Animals and other organisms
☒	☐	Human research participants
☒	☐	Clinical data

Methods

n/a	Involved in the study	
☒	☐	ChIP-seq
☒	☐	Flow cytometry
☒	☐	MRI-based neuroimaging

Animals and other organisms

Policy information about studies involving animals; ARRIVE guidelines recommended for reporting animal research

Laboratory animals	Primary cell cultures of neonatal rat ventricles (Wistar) of wither sex were obtained using published procedures as indicated in the method section.
Wild animals	n/a
Field-collected samples	n/a
Ethics oversight	Experiments were performed following strict federal regulations under license BE27/17 of the State Veterinary Department of the Canton of Bern (indicated in the methods section).

Note that full information on the approval of the study protocol must also be provided in the manuscript.
